# Investigate channel rectifications and neural dynamics by an electrodiffusive Gauss-Nernst-Planck approach

**DOI:** 10.1371/journal.pcbi.1012883

**Published:** 2025-06-30

**Authors:** Zichao Liu, Yinyun Li

**Affiliations:** 1 School of Systems Science, Beijing Normal University, Beijing, China; 2 Computational Neuroscience Unit, Okinawa Institute of Science and Technology, Okinawa, Japan; University of Birmingham, UNITED KINGDOM OF GREAT BRITAIN AND NORTHERN IRELAND

## Abstract

Electrodiffusion plays a crucial role in modulating ion channel conductivity and neural firing dynamics within the nervous system. However, the relationship among ion electrodiffusion, concentration changes, as well as channel conductivity and neuronal discharge behaviors is not quite clear. In this work, we introduce a novel Gauss-Nernst-Planck (GNP) approach to investigate how electrodiffusive dynamics influence ion channel rectification and neural activity. We have analytically demonstrated how the membrane conductance changes along with voltage and ion concentrations due to the electrodiffusive dynamics, bridging the gap between the permeability-based Goldman-Hodgkin-Katz (GHK) model and conductance-based models. We characterize the rectification properties of ${GABA}_{A}$, AMPA and leaky channels by estimating their single-channel permeabilities and conductance. By integrating these rectifying channels into neurodynamic models, our GNP neurodynamic model reveals how electrodiffusive dynamics fundamentally shape neural firing by modulating membrane conductance and the interplay between passive and active ion transport-mechanisms, which exhibits difference from conventional conductance-based neurodynamic models especially when ion concentration accumulates to high levels. Furthermore, we have explored how the electrodiffusive dynamics influence the pathological neural events by modulating the stability of neurodynamic system. This study provides a fundamental mechanistic understanding of electrodiffusion regulation in neural activity and establishes a robust framework for future research in neurophysiology.

## Author summary

Variations in neural membrane potential are driven by transmembrane ion fluxes, which are governed by both the electric potential and ion concentration gradients—a process known as electrodiffusion. Due to this, the conductivities of ion channels are inherently both concentration- and voltage-dependent. However, these intrinsic properties are often ignored in neurodynamic models, such as the traditional Hodgkin-Huxley-like models. In this study, we introduce a Gauss-Nernst-Planck framework that effectively incorporates fundamental electrodiffusive ion transport mechanisms into neurodynamic modeling. We analytically demonstrate that ion channel conductance is determined by both permeability and intramembrane ion concentrations, leading to channel rectification and modification of neural dynamics. We explore how electrodiffusion shapes the rectification behavior of leaky channels, AMPA and ${GABA}_{A}$ receptor channels, and incorporate these rectifying channels into our neurodynamic model. Through comparison with a conductance-based model, we show that electrodiffusion reduces neural excitability under weak glutamate stimulation and enhances the stability of the depolarization block state under strong glutamate stimulation. Our results demonstrate that during intensive firing activities such as epileptic firing or cortical spreading depression with dramatic ion concentration variations, the electrodiffusion effect cannot be ignored.

## Introduction

The transmembrane movement of ions alters the electric potential across the neural membrane, forming the basis of various physiological and pathological neural behaviors [1,2]. Unlike the movement of electrons in conductors, which is solely driven by electric potential differences, ion fluxes in the neural system are influenced by both electric potential and concentration gradients, collectively characterized as “electrodiffusive movement” [3,4]. The unique properties of transmembrane electrodiffusion are captured by the well-known Goldman-Hodgkin-Katz (GHK) current equation, which explains the nonlinear current-voltage (I-V) relationships observed in various ion channels, a phenomenon known as Goldman rectification [5,6]. Researchers have successfully applied electrodiffusive approaches to describe ion transport processes within microscopic compartments of the neural system, such as synaptic clefts [7], dendritic spines [8–11], and ion channels [12–15]. However, at the whole-neuron level, most studies continue to model the neural membrane using simplified electric circuit analogies, leaving the potential influence of electrodiffusive dynamics on neuronal electrophysiology insufficiently explored [16,17].

Although many ion channels exhibit Goldman rectification, conductance-based neurodynamic models—widely used to simulate neural activity under both physiological and pathological conditions—often assume linear I–V relationships for the ion channels they incorporate [18]. This simplification disregards the underlying electrodiffusive processes, even though neural activity fundamentally relies on ion channels exhibiting significant rectification properties. Given that ion channel rectification plays a critical role in shaping neural function [19], its omission in traditional conductance-based models may lead to inaccurate simulations and misinterpretations of neural electrophysiology. This issue becomes particularly concerning in pathological conditions such as epileptic seizures and cortical spreading depression, where ion concentration gradients undergo dramatic shifts [20]. These shifts reshape transmembrane electrodiffusion and alter channel rectification properties. While some researchers have acknowledged the relevance of electrodiffusion under such conditions [21,22], it remains frequently overlooked in related studies. For instance, most computational models of seizure-like activity rely on conductance-based frameworks [23–25], partly due to the challenges of implementing electrodiffusion in whole-neuron simulations.

The questions like how does electrodiffusion-induced channel rectification impact on pathological neural dynamics and how does it deviate from the circuit-based simulations remain largely unknown. Addressing these challenges requires a deeper investigation into transmembrane electrodiffusion and its function on channel rectification during neural discharge activities to refine our understanding of neural function.

However, incorporating rectifying ion channels into neurodynamic models remains a challenging task. More specifically, while many ion channels exhibit rectification properties, others, such as α-Amino-3-hydroxy-5-methyl-4-isoxazolepropionic acid (AMPA) and NALCN channels, display linear I-V relationships [26,27]. The alignment of these linear I-V curves with circuit equations raises questions about whether electrodiffusive dynamics are necessary for modeling currents through such channels. Additionally, certain ion channels, such as Gamma-Aminobutyric Acid Type A (${GABA}_{A}$) receptors, exhibit strong outward rectification under physiological conditions, yet quantifying their single-channel conductance remains difficult [28,29]. Some studies estimate ${GABA}_{A}$ channel conductance by measuring the slope of I-V curves [30], but the validity of this approach is debated, and it is not easily applicable in constructing whole-cell neurodynamic models.

Many studies have investigated electrodiffusion in the nervous system by solving the Poisson-Nernst-Planck (PNP) equations, providing high-resolution simulations of rapid ion transport within small volumes [7–15,31]. However, solving PNP equations requires substantial computational resources, making them impractical for whole-neuron dynamic modeling. Although there have been promising efforts to reduce the computational load of PNP models [32–35], their efficiency still falls short of that of conductance-based models. On the other hand, while the GHK equation effectively captures channel-level rectification induced by electrodiffusion, it does not quantitatively describe how membrane or channel conductance varies with voltage and ion concentrations, limiting its utility in modeling overall electrophysiological behavior. As a result, a practical neurodynamic model that effectively incorporates electrodiffusive dynamics remains lacking.

In this paper, we introduce a novel Gauss-Nernst-Planck (GNP) framework that models the electrodiffusive transmembrane ion dynamics in regulating channel properties and neural dynamics. Our GNP approach reveals the intrinsic quantitative relation between membrane conductivity and permeability, effectively bridging the gap between the permeability-based GHK current equation and the conductance-based circuit models. Using this method, we estimate the permeabilities and conductance of ${GABA}_{A}$ and AMPA channels for their respective permeant ions. We elucidate the mechanisms behind the outward rectification of ${GABA}_{A}$ channels [28–30], the linear I-V relationship of GluR2-containing AMPA channels [26,36], and the inward rectification of GluR2-lacking AMPA channels [37,38]. Additionally, we developed a neurodynamic model incorporating rectifying ion channels to explore how electrodiffusive dynamics fundamentally modulate neural firing activity, and how our model deviates from traditional conductance-based models in simulating neural electrophysiology. We demonstrate that even under negligible ion concentration changes, the electrodiffusion-based model exhibits lower excitability compared to the traditional conductance-based model, due to the inclusion of ion channel rectification. Furthermore, when ion concentrations undergo substantial changes, electrodiffusion enhances passive ion transport across the membrane, thereby promoting the stability of the depolarization block (DB) state in neural systems. We conclude our main results and discuss the potential application of our model in the Discussion.

## Results

### Determine the membrane conductivity through electrodiffusive dynamics

We introduce an innovative approach to integrating intramembrane electrodiffusive dynamics into neural system modeling.

**Fig 1:**
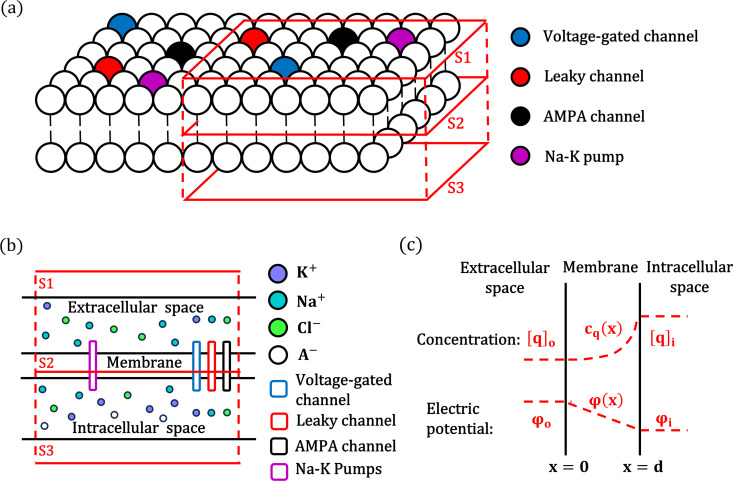
Schematic illustration of the Gauss-Nernst-Planck approach. (a) Neuronal membrane with various channels and $Na^{+}/K^{+}-ATPase$ inserted into the parallel bilayer-lipid structure. Surfaces S1, S2, and S3 are parallel, with S2 located within the intramembrane space. (b) Illustration of space division for the neuron membrane. The intracellular and extracellular spaces contain different ion concentrations ($K^{+},Na^{+},Cl^{-}$), ions move through membrane by electrodiffusion via channels such as leaky channel, voltage-gated channel and also by active transport of $Na^{+}/K^{+}-ATPase$. $A^{-}$ represents impermeable anions confined to the intracellular space. (c) The concentration profile of ions ($c_{q}(x)$) and electric potential distribution (ϕ(x)) in the intramembrane space. The ion concentration profile is nonlinear, while the electric potential profile is linear by Gauss’s law. Extracellular space: x<0, intracellular space: x>d. intramembrane space: x∈[0,d].

By assuming overall electrical neutrality in the neural system (Fig 1a, 1b), we leverage Gauss’s Law to replace the Poisson equation in the Poisson-Nernst-Planck (PNP) framework. This key simplification not only reduces the complexity of the PNP problem but also establishes a crucial link between electrodiffusive- and conductance-based models (see S1 Appendix for details).

Through this Gauss-Nernst-Planck framework, we demonstrate that membrane conductance (per unit area) can be expressed as a function of permeability and the intramembrane concentration profile (denoted as $c_{q}(x)$, see Fig 1c) of the permeant ion. This relationship provides a fundamental link between ion electrodiffusion dynamics and membrane properties, as shown in Eq. 1:


$$G_{q}=\frac{z_{q}^{2}F^{2}}{RT}P_{q}\overset{―}{c_{q}}$$ (1)

**Fig 2:**
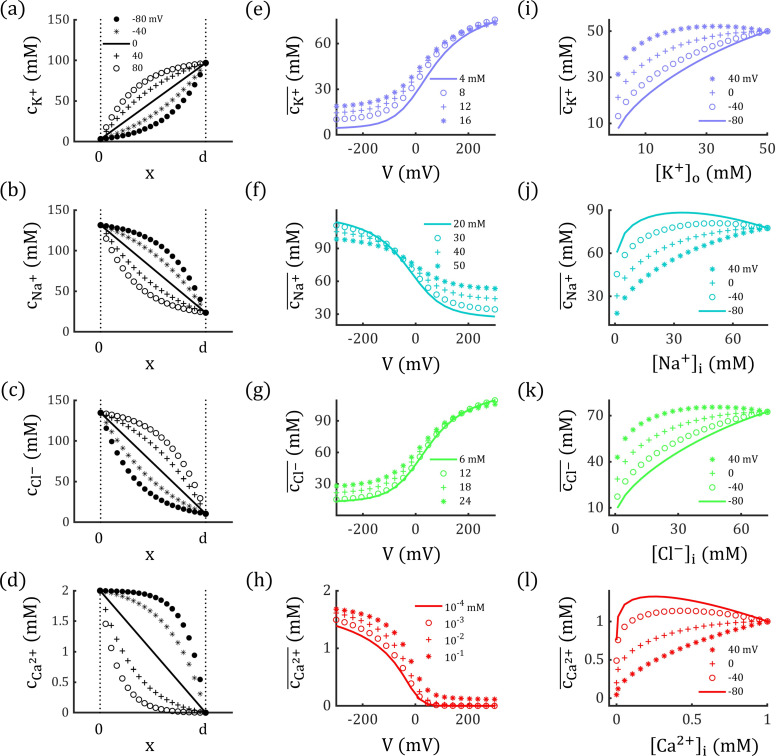
Ion Concentration profiles $c_{q}(x)$ **and corresponding harmonic means** $\overset{―}{c_{q}}\;$**as functions of membrane potential and ion concentrations.** (a-d) Variation of concentration profiles for potassium, sodium, chloride, and calcium ions with different membrane potentials. (e-h) Harmonic mean of each ion concentration profile changes with membrane potential. As membrane potential increases, $\overset{―}{c_{K^{+}}}$ and $\overset{―}{c_{{Cl}^{-}}}$ increase, resulting in outward rectification, whereas $\overset{―}{c_{{Na}^{+}}}$ and $\overset{―}{c_{{Ca}^{2+}}}$ decrease, leading to inward rectification. (i-l) Harmonic means of each ion concentration profile typically increases as the concentration gradient $\Delta c_{q}=|[q{]}_{i}-[q{]}_{o}|$ decreases. The total ion concentration ($[q{]}_{i}+[q{]}_{o}$) is held constant.

Where, R is the gas constant, T is temperature, $P_{q}$ is the permeability of channel to ion q, and $\overset{―}{c_{q}}$ is the harmonic mean of intramembrane concentration profile $c_{q}(x)$. Eq. 1 indicates that higher permeability and ion concentration lead to higher membrane conductivity. At steady state, $c_{q}(x)$ is determined by both ion concentrations and membrane potential (see Eq. 13). As shown in Fig 2a-2d, variations in membrane potential result in changes in the concentration profiles of potassium, sodium, chloride, and calcium ions, which subsequently alter membrane conductance and induce Goldman rectification.

Importantly, Eq. 1 bridges the conductance-based model and permeability-based electrodiffusive approach [39], which allows us to model neural firing activity by both circuit-like and GHK current equations through GNP approach, and investigate the effect of electrodiffusion on neural firing properties (see Methods).

To further illustrate how concentration profiles influence conductivity, we derived an analytical expression for $\overset{―}{c_{q}}$ by integrating $c_{q}(x)$ (see Methods):


$$\overset{―}{c_{q}}=\frac{d}{\int_{0}^{d}\frac{1}{c_{q}(x)}dx}=\frac{[q{]}_{i}-[q{]}_{o}e^{-\frac{z_{q}FV}{RT}}}{1-e^{-\frac{z_{q}FV}{RT}}}\frac{V}{V-\frac{RT}{z_{q}F}\ln\left(\frac{[q{]}_{o}}{[q{]}_{i}}\right)}$$ (2)

Here $\overset{―}{c_{q}}$ represents the mean value of intramembrane ion concentration, which is derived by combining Nernst-Planck equation and the overall electroneutrality assumption (see Methods). Eq. 2 highlights that both membrane potential and ion concentrations affect $\overset{―}{c_{q}}$, thereby altering conductance by Eq. 1. As shown in Fig 2e–2h, the harmonic means of the concentration profiles for potassium and chloride ions $\overset{―}{c_{K^{+}}}$ and $\overset{―}{c_{{Cl}^{-}}}$ increase with membrane potentials, whereas $\overset{―}{c_{{Na}^{+}}}$ and $\overset{―}{c_{{Ca}^{2+}}}$ decrease with it. These shifts lead to higher conductance for inhibitory currents and lower conductance for excitatory currents during depolarization. This differential conductance produces outward rectification for potassium and chloride currents and inward rectification for sodium and calcium currents. The voltage dependent properties suggest dynamic alterations of membrane potential will also modify the channel conductance by altering $\overset{―}{c_{q}}$, creating a feedback loop that is absent in traditional conductance-based neurodynamic models.

Moreover, as illustrated in Fig 2i–2l, $\overset{―}{c_{q}}$ typically increases as the concentration gradient $\Delta c_{q}=|[q{]}_{i}-[q{]}_{o}|$ decreases, which subsequently upregulates the associated conductance. The variation of $\overset{―}{c_{q}}$ also diminishes with decreasing $\Delta c_{q}$, reducing rectification and eventually producing a linear I-V relation when $\Delta c_{q}=0\;mM$. During physiologically normal neural activity, homeostatic mechanisms help stabilize $\Delta c_{q}$ [40], which may explain why the concentration-dependence of membrane conductance is often overlooked. However, under pathological conditions like depolarization blocks, $\Delta c_{q}$ can change significantly [41–42], which would be illustrated in the following section.

### Characterizing channel rectification by GNP approach

The GHK current equation has been widely applied to investigate the biophysical and electrical properties of ion channels, particularly because many channels exhibit Goldman rectification. However, without accurately characterizing the permeability and conductivity of channels, the application of their rectification properties in neurodynamic models remains challenging.

Some of the key unresolved questions include:

1. Conductance of rectifying channel: Many channels, such as ${GABA}_{A}$ channels [28–30], display rectification, rendering their voltage-dependent conductance difficult to be accurately determined. While some studies derive the conductance by taking the slope of the I-V curve (the derivative of the GHK current equation with respect to voltage), this method is neither effective nor precise.
2. Single channel permeability: The single-channel conductance of ion channels is measured through their linear I-V curve, such as AMPA channels [26,36]. However, as shown in Eq. 2, the conductivity is influenced by various factors, making it less informative to characterize the channel’s intrinsic biophysical properties than channel permeability. However, channel permeabilities for different ions are rarely measured.
3. Mechanisms of rectification: Channels like AMPA and NALCN typically exhibit a linear I-V relationship [26,27,36]. However, under certain conditions, such as AMPA channels lack GluR2 subunit and ion concentrations are changed around NALCN channels, these channels could show rectification [27,37,38]. The underlying mechanism driving this shift remains unclear.
4. Specifying ionic currents: Many ion channels are permeable to multiple ion types. While total current is easily measurable, separating the ionic currents for each ion type is challenging. It is often assumed that the respective ionic currents are proportional to permeability ratios [26,29], but this assumption is problematic due to the non-linear functions revealed by Eqs. 1, 2.

To address those challenges, we propose a practical methodology to determine the conductivity and permeability of ion channels based on available data. First, we determine how to achieve a linear I-V relationship by adjusting ion concentrations in individual ion channels (see Eq. 15 in Methods). Next, we calculate single-channel permeabilities using single-channel conductance (the slope of the linear I-V curve) and ion concentration data (see Eq. 16 in Methods). Finally, we quantify channel conductance for both specific permeant ion currents and total current (see Eqs. 18–25 in Methods). We will use this framework to address the issues mentioned above, by taking ${GABA}_{A}$ channel and AMPA channel as examples.

### ${GABA}_{A}$ Channel

To apply the GNP framework to analyze channel rectification properties, the required information includes permeability ratios between different permeant ions, single-channel conductance, the pore diameter of the channel, and permeants’ concentrations in the intra- and extracellular spaces. All those data have been reported in previous experimental research.

For ${GABA}_{A}$ channel, both chloride and bicarbonate ions as permeants with valence of −1. Experimental studies suggest that the permeability ratio $P_{HCO_{3}^{-}}/P_{Cl^{-}}$ is approximately 0.2, and the pore diameter of the channel is around 5 Α [29,30]. The concentrations of $HCO_{3}^{-}$ ions are set at ${\left[HCO_{3}^{-}\right]}_{i}=15\;mM$, ${\left[HCO_{3}^{-}\right]}_{o}=25\;mM$ [40].

In experiments, to measure the single-channel conductance of ${GABA}_{A}$ channel, both ${\left[Cl^{-}\right]}_{i}$ and ${\left[Cl^{-}\right]}_{o}$ are elevated to about 145 mM to eliminate Goldman rectification [29]. According to our GNP framework, to ensure a strictly linear I-V relationship, ${\left[Cl^{-}\right]}_{i}$ should be 2 mM higher than ${\left[Cl^{-}\right]}_{o}$, given that $HCO_{3}^{-}$ ions have asymmetric concentrations. Thus, we assume ${\left[Cl^{-}\right]}_{i}=147\;mM$ and ${\left[Cl^{-}\right]}_{o}=145\;mM$. Under similar conditions, experimental studies report the single-channel conductance of ${GABA}_{A}$ channels to be around 30 pS [30,43]. Based on these data, we can calculate out the permeabilities of ${GABA}_{A}$ channels for chloride ion $P_{Cl^{-}}=7.04\times 10^{-2}\;m/s$ and for bicarbonate ion $P_{{HCO}_{3}^{-}}=1.41\times 10^{-2}\;m/s$ (refers to Eq. 16).

**Fig 3:**
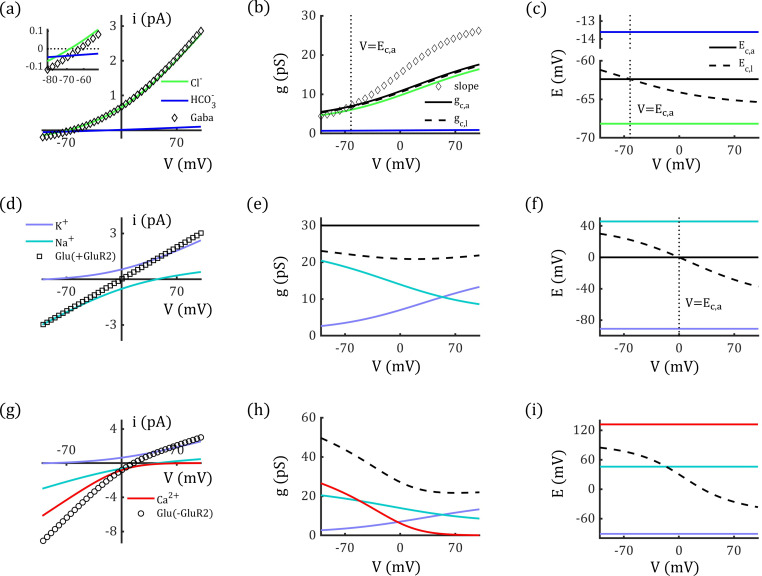
Rectification of $GABA_{A}$ **and** AMPA **channels.** (a-c) Outward rectification of single $GABA_{A}$ channel (a), corresponding conductance (b), and reversal potentials (c). Inset figure: curves are zoomed in around the reversal potential. (d-f) Linear I-V relation of single GluR2-containing AMPA channel which is impermeable to calcium ions, corresponding conductance (e), and reversal potentials (f). (g-i) Inward Rectification of single GluR2-lacking AMPA channel which is permeable to calcium ions, corresponding conductance (h), and reversal potentials (i). Here the calcium concentrations are set to be ${\left[Ca^{+}\right]}_{o}=2\;mM$ and ${\left[Ca^{+}\right]}_{i}=1\times 10^{-4}\;mM$, and the permeability is $P_{{Ca}^{2+}}=2.70\;m/s$. In (b, e): Two methods are used to calculate single channel conductance: $g_{c,a}$ (solid black line) and $g_{c,l}$ (dashed black line). In (c, f): Two methods are used to represent the channels’ reversal potentials: $E_{c,a}$ (solid black line) and $E_{c,l}$ (dashed black line). In (h, i): $g_{c,a}$ and $E_{c,a}$ are not applicable to the GluR2-lacking AMPA channel which is permeable to calcium ions, as calcium has different valence from potassium and sodium ions, whereas, $g_{c,l}$ and $E_{c,l}$ are still valid to describe the I-V curve (dashed black line). The diamond curve in (b) represents the slope of the channel’s I-V curve; the vertical dotted lines in (b), (c), and (f) represent $V=E_{c,a}$. The I-V curves represent $-i_{c}$ and $-i_{q}$, respectively, to align with experimental formats.

Under physiological conditions, ${\left[Cl^{-}\right]}_{i}$ is much lower than ${\left[Cl^{-}\right]}_{o}$, which creates outward rectification in ${GABA}_{A}$ channels and voltage-dependent conductance [43]. With the estimated single-channel permeability, we can reproduce the I-V curve of a single ${GABA}_{A}$ channel (Eq. 19) and determine the corresponding conductance (see Eq. 20 in Methods), as shown in Fig 3a (diamond) and 3b (solid black curve). Notably, the conductance calculated using Eq. 20 is not equivalent to the slope of the I-V curve except when the voltage equals the channel’s reversal potential (Fig 3b, dotted line; see also S1 Fig).

Furthermore, using the permeability of each permeant, we can determine the chloride and bicarbonate currents and conductance, as shown in Fig 3a and 3b (colored curves). Notably, the currents and conductance ratios are neither equal to the permeability ratio ($P_{HCO_{3}^{-}}/P_{Cl^{-}}$) nor constant.

### AMPA Channel

As another example, we investigated the properties of AMPA channels, which are permeable to both potassium and sodium ions. The corresponding concentrations are assumed to be ${\left[K^{+}\right]}_{i}=96.83\;mM$, ${\left[K^{+}\right]}_{o}=3.17\;mM$, ${\left[{Na}^{+}\right]}_{i}=23.58\;mM$, ${\left[{Na}^{+}\right]}_{o}=131.42\;mM$ [40]. For these concentrations, the permeability ratio $P_{Na^{+}}/P_{K^{+}}$ needs to be 0.87 in AMPA channels to ensure exact linear I-V relationship. Based on experimental data [36,44], the single-channel conductance and pore diameter of AMPA channels are comparable to those of ${GABA}_{A}$ channels (30 pS, 5 Α). Using Eq. 16, the permeabilities of AMPA channels are estimated to be $P_{K^{+}}=8.99\times 10^{-2}\;m/s$ and $P_{Na^{+}}=7.80\times 10^{-2}\;m/s$.

We reproduced the linear I-V curve for an individual AMPA channel using the estimated permeabilities, as shown in Fig 3d (square). AMPA channels are typically characterized by a linear I-V relationship and are generally considered non-rectifying. However, as shown in Fig 3d, our results suggest that the apparent linearity of AMPA currents may result from a counterbalance between the outward rectification of potassium currents and the inward rectification of sodium currents. This implies that rectification would naturally arise in AMPA channels if the counterbalance is disrupted due to changes in ion concentrations or permeability ratio. The conductance of potassium and sodium currents are illustrated in Fig 3e (colored curves). Again, the ratios of currents and conductance do not equal the ratio of permeability, nor are they constant.

AMPA channels exhibit inward rectification when they have high calcium permeability due to the absence of the GluR2 subunit [37]. GluR2-lacking AMPA channels are highly permeable to calcium ions leading to a calcium current that exhibits strong inward rectification. As shown in Fig 3g, the emergence of a calcium component (with conductance comparable to potassium and sodium currents, see Fig 3h) disrupts the balance between potassium and sodium currents, ultimately causing the channel to display inward rectification. While substantial evidence suggests that this rectification is primarily due to polyamine block, the contribution of calcium currents remains unclear. Further investigation is needed to fully understand this phenomenon, including studies in calcium-free conditions to isolate the influence of calcium currents.

Notably, the “apparent” conductance $g_{c,a}$ of $Ca^{2+}$-permeable AMPA channels (see Eq. 20 and black solid curves in Fig 3b, e) cannot be calculated in the same way as for ${GABA}_{A}$ and GluR2-containing AMPA channels, since calcium ions have a different valence compared to sodium and potassium ions. Interestingly, an alternative conductance (named as “latent” conductance) formulation (see Eq. 24) effectively characterizes these channels and remains valid for channels with different permeant valences. This “latent” conductance $g_{c,l}$ is represented by black dashed curves in Fig 3b, 3e, and 3h, while the corresponding latent reversal potentials $E_{c,l}$ are shown as black dashed curves in Fig 3c, 3f, and 3i.

Importantly, both latent conductance and reversal potential are voltage-dependent, even for channels with linear I-V relationships. The apparent conductance $g_{c,a}$ and reversal potential $E_{c,a}$ should always be paired with the latent conductance $g_{c,l}$ and latent reversal potential $E_{c,l}$ when describing channel properties. However, previous studies have often mismatched these parameters. A detailed discussion of apparent and latent conductance and reversal potentials is provided in the Methods and S2 Appendix.

### Modeling neural dynamics by GNP approach

The results presented in the previous sections were obtained under steady-state conditions (see Methods). To further incorporate the dynamics of electrodiffusive ion transport into neural firing dynamics, such as neural discharges, we assume that the electrodiffusive dynamics within ion channels are much faster than the voltage dynamics (see S3 Appendix Quasi-Static Assumption).

**Fig 4:**
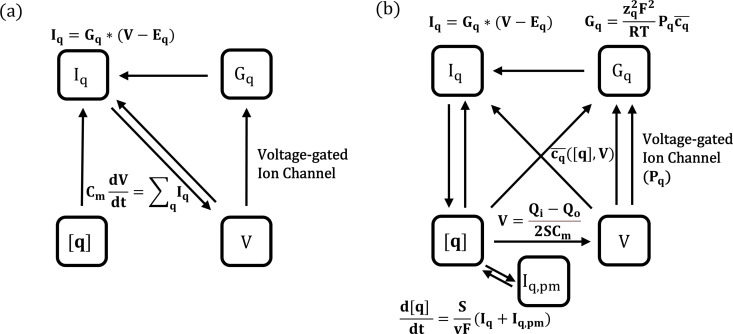
Comparison between classical equivalent-circuit-based model and our GNP model. Panels (a) and (b) depict the dynamic schematics of the classical equivalent-circuit-based model and our electrodiffusion-based GNP model, respectively. $I_{q}$: electrodiffusive ion current through ion channels; $I_{q,pm}$: current induced by $Na^{+}/K^{+}-ATPase\;$pumps; V: membrane potential; [q]: ion concentrations; $G_{q}$: membrane conductance per unit area; $Q_{i/o}$: net charge in intra- and extracellular spaces; $\overset{―}{c_{q}}$: harmonic mean of intramembrane concentration profile.

Thereafter, we build a neural dynamics model based on our GNP method, where, the electrodiffusive ion transport would interact with ion concentration changes and membrane potential. The changes of ion concentrations and membrane potential would in turn modulate the electrodiffusive dynamics, forming a closed feedback loop, as shown in Fig 4. Our GNP approach explicitly incorporates electrodiffusive dynamics by modifying membrane conductance $G_{q}$, enabling a more comprehensive neurodynamic model that accounts for rectifying channels (Fig 4b). Moreover, the GNP model captures the direct connection between channel rectification induced by electrodiffusion and neural activity, highlighting the role of electrodiffusion in passive-active ion transport interactions which shapes neural stability. The simulation results and analysis by this model are displayed in following sections.

### Outwardly rectifying leaky current enhance neural stability

**Fig 5:**
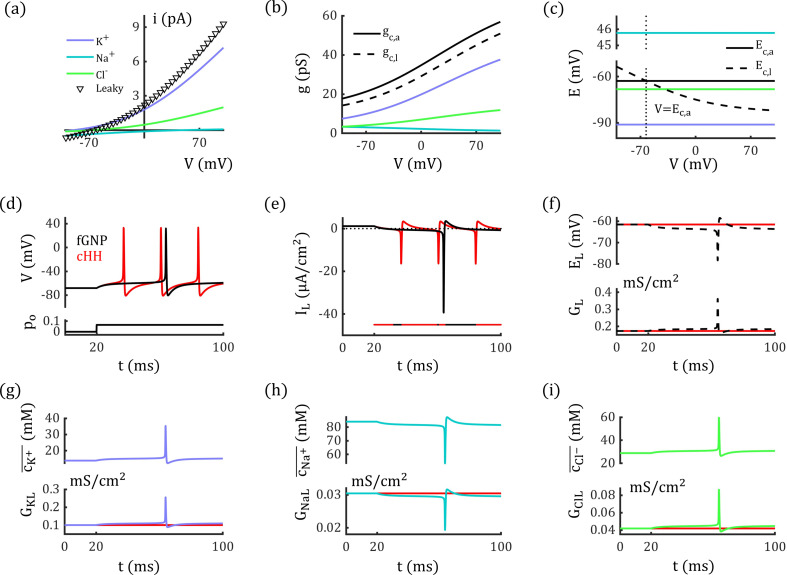
Outward rectification of leaky channels enhances neural stability. (a) The outward rectification of single leaky channel (triangle) used in fGNP model, where, each ion current is shown in purple ($K^{+}$), cyan ($Na^{+}$) and green ($Cl^{-}$) curves. (b) Conductance of leaky channel as function of voltage. Solid black line represents $G_{c,a}$, dashed black line represents $G_{c,l}$, colored lines represent the conductance of respective ions. (c) Reversal potential of the leaky channel. Solid black line represents $E_{c,a}$, dashed black line represents $E_{c,l}$, colored lines represent reversal potentials of respective ions. (d) The neuron in the fGNP model generates fewer action potentials than the cHH model with same tonic glutamate stimuli. Black line represents concentration-fixed GNP (fGNP) model with rectifying leaky channels, red line represents the classical HH-type (cHH) model with linear leaky channel. (e) Leaky currents in the fGNP and cHH models during stimulation, with the horizontal line indicating the model with higher leaky current at each time point. The dotted line represents $0\;\mu A/cm^{2}$. (f) Conductance of the leaky channel in the fGNP model. Dashed black lines represent $G_{c,l}$ and $E_{c,l}$ in fGNP model, red lines represent constant $G_{LH}$ and $E_{LH}$ in cHH model. (g-i) Harmonic mean and conductance of potassium and chloride ions increase, for sodium ions they decrease accompanying with the depolarization.

Leaky currents arise from various ion channels within the membrane [27,45–47]. In our model, we simplify these currents as an equivalent “leaky channel” characterized by high potassium permeability ($P_{K^{+}}=2.55\times 10^{-1}\;m/s$), medium chloride permeability ($P_{{Cl}^{-}}=5.08\times 10^{-2}\;m/s$), and low sodium permeability ($P_{{Na}^{+}}=1.27\times 10^{-2}\;m/s$). This leaky channel exhibits outward rectification, as shown in Fig 5a-c.

Unlike the classical Hodgkin-Huxley (cHH) models [48], which assume constant conductance of leaky channel, our GNP model incorporates outwardly rectifying leaky currents. To explicitly see the effect of this rectification on neural dynamics, we fix ion concentrations in the GNP model to steady state (see Tab. 1) and compare the neural behavior in this concentration-fixed GNP (fGNP) model to that in the cHH model (see Concentration-Fixed GNP Model in Methods). To determine the average membrane permeability, we assume there are $1\times 10^{7}$ leaky channels and $4\times 10^{6}$ AMPA channels (see Fig 3a) per square centimeter of membrane [17,49], with an opening probability ($p_{o}$) of AMPA channels to represent the intensity of glutamate stimuli. Voltage-gated currents are modeled using reduced Traub-Miles (RTM) equations (see Methods) [50].

As shown in Fig 5d, the fGNP and cHH models respond differently to identical glutamate stimuli. With $p_{o}$ increasing from 0 to 0.065, the neuron in the fGNP model fires only one action potential within 80 ms, while the neuron in the cHH model fires three. The voltage-dependence of rectifying leaky channels lead to the lower excitability in fGNP model. As shown in Fig 5g-i, when glutamate stimuli evoke depolarization response, the leaky conductance for potassium and chloride ions increases, while the conductance for sodium ions decreases, which would enhance the inhibitory potassium and chloride currents and weaken the excitatory sodium current. Those changes cause the overall leaky conductance $G_{L,l}$ increases, while the leaky reversal potential $G_{L,l}$ decreases in fGNP model (Fig 5f). As a result, the overall leaky current in the fGNP model is typically lower than in the cHH model (Fig 5e), thus reduces neural excitability.

### Electrodiffusion promotes the interplay between Ion accumulation and neural discharge events

**Fig 6:**
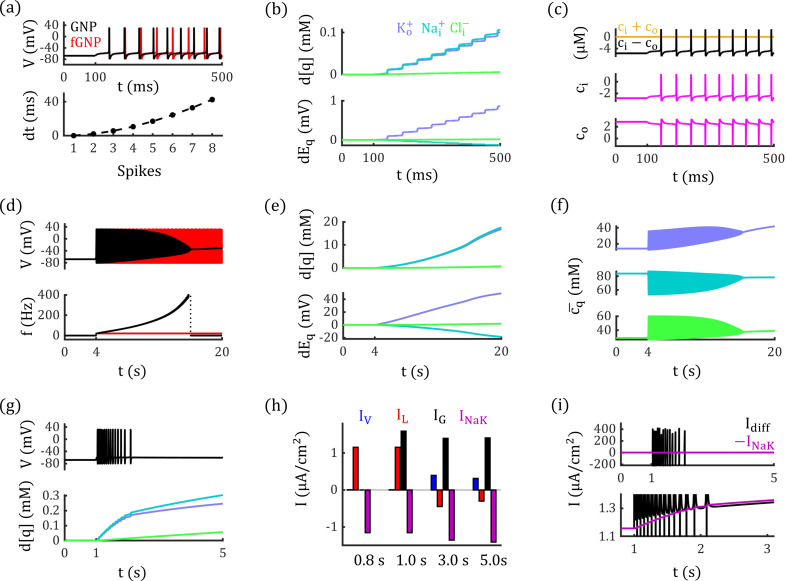
Differential neural dynamics in the GNP model incorporating electrodiffusion and ion concentration changes. (a-f): Constant $Na^{+}/K^{+}-ATPase$ activity. (a) Upper panel: Neural discharge events in response to glutamate stimuli ($p_{o}=0.065$) in the GNP (black) and fGNP (red) models. Lower panel: The timing of discharge events in the GNP model progressively advances the spikes in fGNP model. (b) The deviations of ion concentrations from their steady-state values gradually increase during neural activity (Upper panel), resulting in rising potassium and chloride equilibrium potentials and decreasing sodium equilibrium potential (Lower panel). (c) Dynamics of net charge concentrations in the intra- and extracellular spaces ($c_{i}$ and $c_{o}$ magenta lines); their summation and difference are displayed in the upper panel. (d) In the GNP model (black), the firing frequency progressively increases, eventually leading to depolarization block under prolonged stimuli ($p_{o}=0.065$). In contrast, the neuron in the fGNP model (red) maintains a constant firing frequency under the same stimuli. (e) Significant changes in ion concentrations and reversal potentials occur under prolonged stimulation, following the same trend as shown in (b). (f) The harmonic means of intramembrane potassium and chloride concentrations increase, whereas for sodium, it decreases during neural activity. (g-l): ion-concentration dependent $Na^{+}/K^{+}-ATPase$ activity. (g) With concentration-dependent $Na^{+}/K^{+}-ATPase$ activity, the neuron in the GNP model returns to a resting state after a few action potentials under glutamate stimulation ($p_{o}=0.065$). (h) Currents from voltage-gated channels ($I_{V}$), leaky channels ($I_{L}$), glutamate channels ($I_{G}$), and $Na^{+}/K^{+}-ATPase$ ($I_{NaK}$) at different time points. (i) The $Na^{+}/K^{+}-ATPase$ current (purple) increases, counterbalancing the electrodiffusive current ($I_{diff}$), thereby restoring neural stability.

When incorporating ion concentration dynamics, our GNP model uncovers that subtle ion concentration changes can evoke remarkable voltage signals. As illustrated in Fig 6a, 6b, under tonic glutamate stimulation with $p_{o}=0.065$, ion concentrations in the GNP model are changed due to enhanced electrodiffusive ion transport, leading to a sequence of action potentials. Interestingly, despite significant variations in membrane potential, the corresponding ion concentration changes remain surprisingly small, with all changes below 0.1 mM after nine spikes (Fig 6b).

Notably, the concentrations of net charges in intra- and extracellular spaces ($c_{i}$ and $c_{o}$) are on the micromolar scale (Fig 6c), which are much smaller than the millimolar scale of ion concentrations. As a result, even seemingly negligible ion concentration changes are sufficient to alter the charge difference significantly, thereby dramatically impact neural spike timing. As demonstrated in Fig 6a, the timing of discharge events in the GNP model (black line) deviates significantly from the concentration-fixed model (red line) after just nine spikes. Such deviations could influence the neural temporal coding and impact on neural functions [51].

It is important to note that the ion concentration accumulation contributes to the increased neural excitability. As shown in Fig 6a, under tonic glutamate stimulation ($p_{o}=0.065$), the enhanced electrodiffusive ion transport gradually advances the timing of discharge events in the GNP model compared to the fGNP model. With prolonged glutamate stimulation of the same strength, these currents further elevate the firing frequency, ultimately drives the neuron into a depolarization block (DB) state (Fig 6d black curve), while the identical stimuli elicit a constant firing activity in the fGNP model (Fig 6d red curve).

Furthermore, before entering the DB state, both firing frequency and ion concentrations accelerate coherently in GNP model, indicating a positive feedback loop between ion accumulation and neural discharge events. Although changes in conductance and the sodium reversal potential tend to reduce excitability (Fig 6e, 6f), this feedback loop is primarily driven by the elevation of potassium reversal potentials (Fig 6e), which significantly weakens the inhibitory effects of potassium and chloride currents.

### Interplay of electrodiffusive and active ion transports determines neurodynamic state

In Fig 6d, the neuron generates high-frequency spiking and eventually enters the DB state. This transition occurs because the external glutamate stimuli disrupt the initial balance between passive ion transport by electrodiffusion and active ion transport by $Na^{+}/K^{+}-ATPase$ activity, which destroys the neural stability and lead to excessive excitability. To restore stability, the activity of $Na^{+}/K^{+}-ATPase$ needs to increase during neural activity to prevent excessive excitability and maintain normal function. Experimental studies have shown that $Na^{+}/K^{+}-ATPase$ activity is enhanced when extracellular potassium ${\left[K^{+}\right]}_{o}$ and intracellular sodium ${\left[{Na}^{+}\right]}_{i}$ levels rise [52]. To model this process, we implement concentration-dependent $Na^{+}/K^{+}-ATPase$ based on previous research (see Methods) [24,25].

As illustrated in Fig 6g, introducing concentration-dependent $Na^{+}/K^{+}-ATPase$ allows the neuron in the GNP model to regain a resting state after generating several action potentials by glutamate stimuli ($p_{o}=0.065$). During this phasic-spiking event, $Na^{+}/K^{+}-ATPase$ activity increases by approximately 20 %, with the pump-induced current rising from $-1.16\;\mu A/{cm}^{2}$ at t=1 s to $-1.41\;\mu A/{cm}^{2}$ at t=5 s (Fig 6h). This increased $Na^{+}/K^{+}-ATPase$ activity counterbalance the electrodiffusive currents (Fig 6i), eventually results in a resting state.

**Fig 7:**
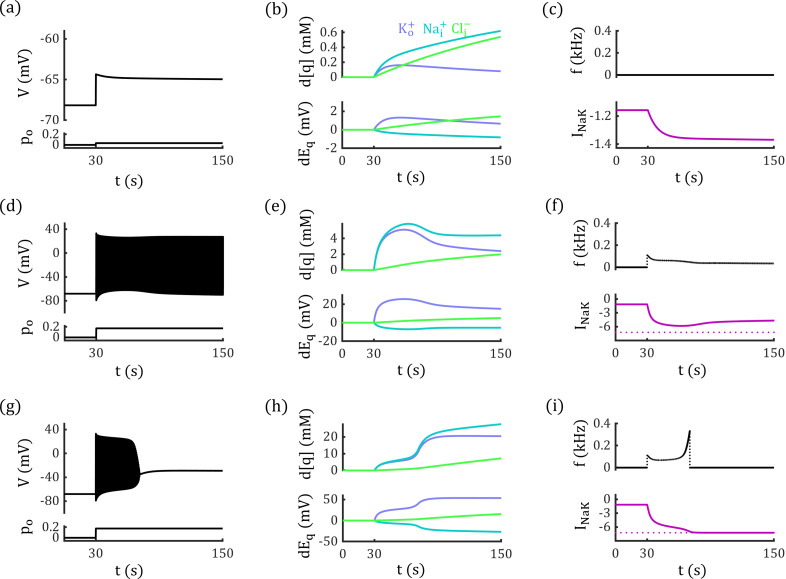
Competition between passive and active transport settles neural activities. (a-c) At $p_{o}=0.033$, the glutamate input enhances passive transport and induces depolarization, but the subsequent increase in $Na^{+}/K^{+}-ATPase$ suppress the passive transport, maintaining resting state. (d-f) At $p_{o}=0.17$, the passive electrodiffusive current and active $Na^{+}/K^{+}-ATPase$ activity reach a dynamic equilibrium, resulting in tonic spiking behavior. (g-i) At $p_{o}=0.18$, the electrodiffusive current overwhelms $Na^{+}/K^{+}-ATPase$ activity, leading the neuron into a DB state. Left column: The glutamate input and the neural activity; Middle column: Changes in ion concentrations and corresponding reversal potentials. Right column: Neural firing frequencies and the changes in pump-induced currents.

The response of $Na^{+}/K^{+}-ATPase$ activity to ion concentration changes influences neural dynamics under tonic glutamate stimulation. In addition to the phasic-spiking event shown in Fig 6g, we identified three other types of neural behaviors arising from the interplay between passive and active ion transport. As shown in Fig 7a, with relatively weak glutamate stimulation at $p_{o}=0.033$, the neuron undergoes slight depolarization. However, the depolarization amplitude gradually decreases following stimulus onset.

With stronger glutamate stimuli at $p_{o}=0.17$, the $Na^{+}/K^{+}-ATPase$ activity and electrodiffusive currents achieve a dynamic equilibrium, resulting in a tonic-spiking state (Fig 7d-7f). As shown in Fig 7d, under $p_{o}=0.17$, neural discharge increases ${\left[K^{+}\right]}_{o}$ and ${\left[{Na}^{+}\right]}_{i}$, which in turn enhances the pump current $I_{NaK}$ (Fig 7e, 7f). The elevated $I_{NaK}$ reduces the neural firing frequency (Fig 7f) and gradually decreases ${\left[K^{+}\right]}_{o}$ and ${\left[{Na}^{+}\right]}_{i}$, counterbalancing the passive electrodiffusive currents and leading to a stable tonic-spiking state.

When the stimulus intensity is further increased to $p_{o}=0.18$, the electrodiffusive currents become too strong to be suppressed by the $Na^{+}/K^{+}-ATPase$ activity, eventually pushing the neuron into the DB state (Fig 7g-7i). As shown in Fig 7i, the firing frequency initially decreases due to the enhanced pump current $I_{NaK}$. However, the upper limit of pump strength makes $I_{NaK}$ fail to counterbalance the enhanced passive transport, and eventually leads the neuron to the DB state.

### Electrodiffusive dynamics modulate the voltage-gate currents and neural dynamics

Traditionally, the dynamic equations governing the gating variables of voltage-gated channels are fit into conductance-based circuit equations ($I_{qV}$) [48,50], whereas gating equations for permeability-based GHK format remain underdeveloped. In above-mentioned GNP model, we simply employed the original RTM equations and ignored the electrodiffusive dynamics within voltage-gated channels. This approach is only valid under minor variations in ion concentrations, as the voltage-dependent conductance changes can be sufficiently captured by the gating equations. However, it may reduce accuracy when modeling neural activities with significant concentration changes, as the concentration-dependence of conductance is overlooked in original RTM equations.

**Fig 8:**
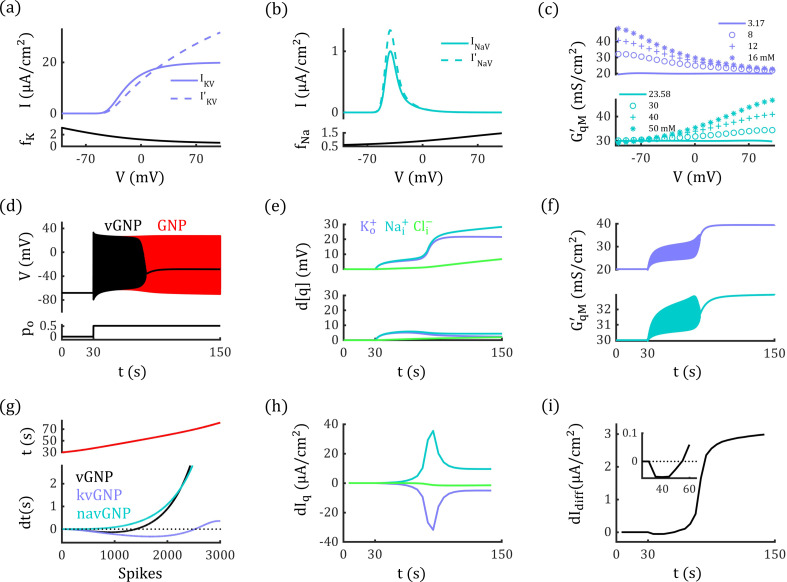
Electrodiffusive voltage-gated currents modulate neural activity. (a) and (b): Upper panel: I-V curves for voltage-gated currents in GHK format ($I_{qV}^{\prime }$) and RTM format ($I_{qV}$) with ion concentrations fixed at steady-state levels. Lower panel: The ratios of $I_{qV}/{I^{\prime }}_{qV}$, which are fitted by function $f_{qV}(V)$ (see Methods). (c) Equivalent maximum conductance of voltage-gated potassium and sodium currents in the vGNP model. (d) Neuron enters the depolarization block state in the vGNP model (black line), while the neuron in the GNP model (red line) shows tonic-spiking by the identical glutamate stimuli ($p_{o}=0.17$). $Na^{+}/K^{+}-ATPase$ strength is concentration-dependent in both models. (e) Deviations in ion concentrations from their steady-state values in the vGNP model (upper panel) and GNP model (lower panel). (f) Maximum conductance of voltage-gated potassium and sodium currents both increases during the simulation in vGNP model.(g) Timing differences in neural discharge events in vGNP (black), kvGNP (purple), navGNP (cyan) models relative to the GNP model, illustrating the lag and lead in action potential generation. Upper panel: Timing of discharge events in the GNP model. (h) Differences of electrodiffusive potassium, sodium, and chloride currents between the vGNP and GNP models. (i) Differences of total electrodiffusive current between the vGNP and GNP models. In (a) and (b), the fitted parameter values are: $\left[N_{K3},N_{K2},N_{K1},N_{K0}\right]=[-1.20\times 10^{-7},1.18\times 10^{-5},0.0075,\;0.85]$ and $\left[N_{Na3},N_{Na2},N_{Na1},N_{Na0}\right]=[9.41\times 10^{-8},4.64\times 10^{-6},-0.0056,\;1.11]$ (see Eq. 43). Data in (h) and (i) are processed using a sliding window (see Methods).

To investigate the potential impact of electrodiffusive dynamics on voltage-gated currents and neural dynamics, we model the voltage-gated currents using the GHK format (${I^{\prime }}_{qV}$) (see Methods). The I-V curves of ${I^{\prime }}_{qV}$ in GHK format differ from those in the RTM model. To align them in steady state, we introduce a function $f_{q}(V)$ to fit $I_{qV}^{\prime }/I_{qV}$, as illustrated in Fig 8a, 8b. After modified by $f_{q}(V)$, the equivalent maximum conductance of the voltage-gated channels ${G^{\prime }}_{KM}$ and ${G^{\prime }}_{NaM}$ exhibits both voltage- and concentration-dependencies, as depicted in Fig 8c.

We incorporate ${I^{\prime }}_{qV}$ into the GNP model and named it as the vGNP model, which shows different neural dynamics from the GNP model. As shown in Fig 8d, with $p_{o}=0.17$, the neuron in the GNP model exhibits tonic-spiking, whereas the neuron in the vGNP model evolves into a DB state. This disparity is due to substantial changes in ion concentrations (Fig 8e), which induce pronounced alterations in the conductance of ${G^{\prime }}_{KM}$ and ${G^{\prime }}_{NaM}$, as demonstrated in Fig 8f.

In Fig 8g, the neural discharge events in the vGNP model initially lag behind those in the GNP model because enhanced potassium conductance leads to stronger inhibition. However, by approximately 50 s, the discharge events in the vGNP model catch up with the GNP model as enhanced sodium conductance becomes dominant. This interplay between enhanced potassium and sodium conductance is further elucidated through the difference of each ion current (Fig 8h) and total electrodiffusive currents (Fig 8i).

Interestingly, in a kvGNP model (incorporating ${I^{\prime }}_{KV}$ and $I_{NaV}$, see Methods), enhanced inhibitory potassium conductance can paradoxically increase neural excitability during certain periods (Fig 8g). This occurs because the elevated conductance mitigates the inhibitory effects of potassium currents by promoting extracellular potassium accumulation and elevating $E_{K^{+}}$. Similarly, in a navGNP model (incorporating $I_{KV}$ and ${I^{\prime }}_{NaV}$), reduced sodium currents can also be observed in certain scenarios, as illustrated in S3 Fig.

### Electrodiffusion enhance the stability of depolarization block by promoting passive Ion transport.

The simulations shown in Fig 7 indicate that our GNP model can capture the dynamic evolution of ion concentrations and membrane potential during neural activities. During pathological conditions like seizures and cortical spreading depression (CSD), the ion concentration distribution would exhibit significant alterations due to the sustained neural firing [40]. Computational studies have provided strong evidence that such changes in ion concentrations can induce bifurcations in the neurodynamic system—phenomena closely associated with pathological events like seizure onset and the DB state [40–42,53].

Here, we demonstrate how an electrodiffusion-based vGNP model deviates from a conductance-based model in generating distinct bifurcation diagrams and reveal the underlying dynamic mechanisms behind these differences. We use a conductance-based neurodynamic model that includes ion concentration dynamics (the ion-concentration-augmented iHH model, with an extension of the classical Hodgkin–Huxley framework [23–25,54], see Methods) to compare with our vGNP model. In the iHH model, the leaky, glutamatergic, and maximum voltage-gated conductances are fixed to the same steady-state values as those in the vGNP model. The maximum strength of the $Na^{+}/K^{+}-ATPase$ is set to $-16.87\;\mu A/cm^{2}$ ($M_{NaK}=0.70\;mM/s$, see Eq. 31) in both models.

**Fig 9:**
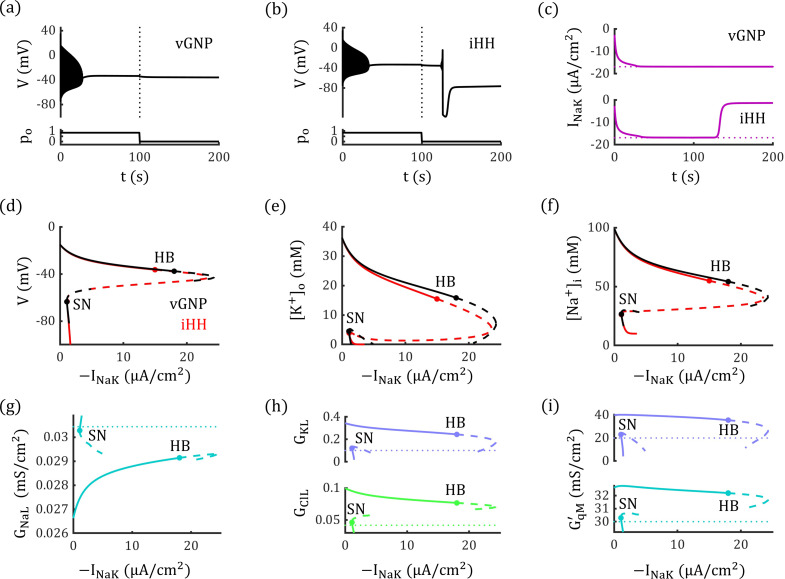
Electrodiffusion enhances passive ion transport and stabilizes the depolarization block state. (a and b) Neurons in both the vGNP and iHH models enter a DB state under glutamate stimulation with $p_{o}=0.83$. Upon removal of the stimulus (indicated by the dotted line), the neuron in the vGNP model remains in the DB state (a), whereas the neuron in the iHH model returns to the resting state (b). (c) Time course of the $Na^{+}/K^{+}-ATPase$ current $I_{NaK}$ in the vGNP (top) and iHH (bottom) models. In both models, $\left|I_{NaK}\right|$ reaches its upper limit (dotted line) during the DB state. This maximum current changes to the termination of the glutamate input and restore the resting state in the iHH model but fails to destabilize the DB state in the vGNP model. (d–f) Bifurcation diagrams of the neurodynamic system as $I_{NaK}$ varies. Both models exhibit saddle-node (SN) and Hopf bifurcations (HB), with the DB state losing stability at the HB point. The HB point occurs at a higher $\left|I_{NaK}\right|$ in the vGNP model than in the iHH model, due to the electrodiffusive dynamics incorporated in the vGNP model. Fixed points in the vGNP model (black curves) form discontinuous branches because, in certain ranges, $I_{NaK}$ drives ${\left[K^{+}\right]}_{o}$ below zero, which is unphysical. (g–i) Membrane conductance at various fixed points in the vGNP model. At fixed points corresponding to the DB state, both leaky potassium and chloride conductance (h), as well as maximum voltage-gated sodium and potassium conductance (i), are higher in the vGNP model compared to the iHH model (dotted lines). These elevated conductances enhance passive ion transport in the vGNP model, necessitating stronger active ion transport to trigger a Hopf bifurcation and destabilize the DB state.

As shown in Fig 9a and 9b, under a glutamate stimulus with $p_{o}=0.83$, neurons in both the vGNP and iHH models enter the DB state. However, after the stimulus is removed, the neuron in the vGNP model maintains a stable DB state, while the neuron in the iHH model returns to the resting state. The stability of the DB state in the vGNP model is supported by stronger passive ion transport, driven by electrodiffusive dynamics that are absent in conductance-based models. To better elucidate this distinction, we performed bifurcation analysis on both the vGNP and iHH models, examined how variations in the $Na^{+}/K^{+}-ATPase$ current $I_{NaK}$ induce bifurcations in both models. Both models exhibit saddle-node (SN) and Hopf bifurcations (HB), with the DB state losing stability at the HB point. However, the HB point occurs at a higher $\left|I_{NaK}\right|$ in the vGNP model than in the iHH model, due to the electrodiffusive dynamics incorporated in the vGNP model (Fig 9d-f).

In Fig 9d–f, we observe that the system can keep a stable fixed point at a saddle-node bifurcation under relatively low $\left|I_{NaK}\right|$. These fixed points correspond to resting states where ion concentrations and membrane potential are close to physiological values. Under such conditions, lower ${\left[K^{+}\right]}_{o}$ and ${\left[{Na}^{+}\right]}_{i}$ result in relatively weak active transport ($\left|I_{NaK}\right|$) and preserve the balance between active and passive mechanisms. When subjected to strong external stimulation, the neural membrane potential, ${\left[K^{+}\right]}_{o}$ and ${\left[{Na}^{+}\right]}_{i}$ increase, leading to enhanced active and passive transport. If the stimulation drives the neuron into a DB state, the vGNP model exhibits significantly stronger passive transport than the iHH model, due to its electrodiffusive effect. Consequently, destabilizing the DB state in the vGNP model requires a much stronger active ion transport. If the physiological upper bound of $I_{NaK}$ in the system cannot reach the bifurcation threshold, the neuron remains in a stable DB state even after the stimulus is removed (Fig 9a)—a phenomenon analogous to that observed in CSD conditions [25,42].

The Hopf bifurcation occurs at a higher value of $\left|I_{NaK}\right|$ in the vGNP model because electrodiffusion enhances passive ion transport. As shown in Fig 9d–f, membrane conductance varies across $I_{NaK}$ in the vGNP model, whereas the iHH model maintains constant conductance. Apart from a slightly lower leaky sodium conductance, the vGNP model exhibits significantly higher leaky potassium and chloride conductance, as well as greater maximum conductance for voltage-gated sodium and potassium channels. This increased conductance in the vGNP model necessitates stronger active transport to destabilize the DB state and induce a Hopf bifurcation. Consequently, the bifurcation occurs at $I_{NaK}=-17.83\;\mu A/cm^{2}$ in the vGNP model, compared to $I_{NaK}=-14.94\;\mu A/cm^{2}$ in the iHH model. As a result, when the upper limit of $\left|I_{NaK}\right|$ is set to $16.87\;\mu A/cm^{2}$, the neuron in the iHH model can return to resting state from the DB state (Fig 9b) when glutamatergic stimuli are removed, while the neuron in the vGNP model cannot but stay still at the DB state (Fig 9a).

These findings suggest that the electrodiffusive vGNP model provides a more accurate and mechanistically grounded framework than traditional conductance-based models for investigating pathological neural dynamics.

## Discussion

This study investigates electrodiffusive dynamics of intramembrane ions on channel rectification and neural firing activities by an efficient Gauss-Nernst-Planck computational framework. We have clarified the relationship between conductivity and permeability with intramembrane ion concentration profiles, which makes critical step to bridge the gap between the permeability-based GHK equation and the conductance-based model. Meanwhile, our GNP method provides a practical approach to determine the permeabilities and conductance of ion channels to each permeant ions, demonstrated with ${GABA}_{A}$ and AMPA channels, which reveals that channel rectification arises from the superimposition of individual permeant ion currents. Our results demonstrate that electrodiffusive ion transport leads to rectifying leaky currents and enhances neural stability. We emphasize that the typically overlooked electrodiffusive dynamics can significantly influence neural activity through a feedback loop involving electrodiffusive ion transport, ion concentration changes, and membrane potential, critical in our model but absent in classical models. We also found that the competition between electrodiffusive ion transport and $Na^{+}/K^{+}-ATPase$ activity plays a crucial role in determining neurodynamic states by excitatory stimuli, such as resting, tonic-spiking, and DB states.

Our GNP framework provides promising potential for broad applications. One area of interest is the role of cellular impermeable anions in maintaining concentration homeostasis and neural functions. Traditionally, these anions have been considered important for osmotic pressure regulation [16,55], but their impact on neural discharge events remains largely unexplored. Some studies propose that local impermeable anions, rather than cation-chloride co-transporters, play the primary role in establishing intracellular chloride concentrations [56,57]; however, other researchers argue that this mechanism may not be physically realistic [58,59]. Traditional conductance-based models are insufficient for exploring this problem, as impermeable anions do not generate transmembrane currents. In contrast, our GNP model incorporates impermeable anion concentrations as a critical factor in determining membrane potential (see Eq. 27), providing an appropriate framework to investigate how variations in these anions affect steady-state conditions and modulate neural activity.

Importantly, our model is well-suited for studying neural dynamics during epileptic seizures, which involve complex discharge patterns such as periodic bursting and DB, accompanied with significant ion concentration oscillations [40]. Additionally, some studies suggest that changes in AMPA channel rectification properties can influence seizure generation [60–62]. These dynamic features are challenging for traditional conductance-based models to capture, but our GNP framework—integrating concentration-dependent membrane conductance and the competition between passive and active transport processes—offers a powerful scheme for modeling the neural dynamics during epileptic seizures.

Although we argue that electrodiffusion is a widespread phenomenon in the nervous system—due to the frequent coexistence of concentration and electrical potential gradients across membranes—it may not always be the primary factor modulating neural electrophysiology. For example, inwardly rectifying potassium (Kir) channels, despite being selectively permeable to potassium ions, exhibit inward rectification due to voltage-dependent blockade by intracellular cations [63]. Similarly, complex biophysical mechanisms such as phosphorylation can influence channel rectification properties [64]. In such cases, electrodiffusive dynamics may act as secondary modulators, and the channel behavior cannot be fully captured by our GNP framework.

Additionally, although we have presented some evidence suggesting that the linear I–V relationship of AMPA channels arises from a counterbalance between electrodiffusive potassium and sodium currents, more direct experimental validation is needed. We propose that measuring the I–V characteristics of AMPA channels in potassium-free and sodium-free solutions could provide clearer evidence of whether electrodiffusion plays a primary role in modulating their behavior. If AMPA channels exhibit inward Goldman rectification in potassium-free solution and outward Goldman rectification in sodium-free solution, this would support our hypothesis. Moreover, examining the I–V relationship of GluA2-lacking AMAP channels in a calcium-free solution could help determine whether calcium influx is the primary contributor to the observed inward rectification.

We found the distinct bifurcation diagram between our GNP framework and classic HH-like models, and reveal that interplay between passive and active ion transport can profoundly shape neural activity. Notably, our results show that electrodiffusion in our GNP framework enhances passive ion transport especially when neural activity leads to substantial ion concentration changes, thereby promoting the stability of the depolarization block (DB) state. This offers a novel perspective on the DB state, a hallmark of neurons during CSD [42]. Previous studies have reported that mutations in SCN1A, which increase the excitability of interneurons, can promote rather than suppress CSD—a counterintuitive finding that remains insufficiently understood [25,65–67]. Our study suggests that this may be due to hyperexcitable interneurons increasing chloride conductance in their target neurons, thereby enhancing passive ion transport and stabilizing the DB state. To test this hypothesis, experimental investigations could assess whether increasing ATP availability or blocking specific ion channels disrupts the stability of the DB state.

A simplification in our model is regarding the dynamics of chloride ions. We omit the contributions of cation-chloride cotransporters such as KCC2 and NKCC1 [68,69], as well as GABAergic stimuli, which leaves the leaky chloride current as the sole mechanism modulating chloride concentrations. Consequently, intracellular chloride accumulation is slower than the changes in extracellular potassium and intracellular sodium concentrations during neural activity (Fig 6). However, the regulation of chloride concentrations plays a crucial role in regulating the inhibitory effects of GABAergic signaling [68,69], which is critical for maintaining proper neural function. Even though this simplification reduces the model’s complexity, it provides a concise and general framework that can be applied and extended to specific topics. A more comprehensive version of the model, incorporating the chloride cotransporters and GABAergic stimuli, will be explored in future studies to investigate the mechanism of chloride dynamics in regulating neural physiology.

## Methods

### The Gauss-Nernst-Planck approach

To model electrodiffusion, a common approach is to solve the Poisson-Nernst-Planck (PNP) equation, expressed as:


$$\begin{array}{c} \left\{ \;\;\;\begin{array}{c} \;\frac{dc_{q}}{dt}=-\nabla \cdot \vec{J_{q}}\mathrm{\backslash vspace}1mm\\ \vec{J_{q}}=-D_{q}\left(\nabla c_{q}+\frac{z_{q}F}{RT}c_{q}\nabla \varphi \right)\mathrm{\backslash vspace}0.5mm\\ \;\nabla^{2}\varphi =-\frac{\rho }{\in_{0}\in_{r}} \end{array}\right.\; \end{array}$$ (3)

Here, $c_{q}$, ϕ and ρ represent the spatial distributions of ion concentration, electric potential, and charge density, respectively. The vector field $\vec{J_{q}}$ denotes the ion flux, while $D_{q}$ is the diffusion coefficient. The term $\in_{0}\in_{r}$ represents the permittivity. The subscript q refers to ion species that permeate the neural membrane.

Solving the PNP equations is computationally demanding. Consequently, neural membranes are often approximated using an equivalent circuit model, described by the following equation:


$$C_{m}\frac{dV}{dt}=\sum_{q}I_{q}=-\sum_{q}G_{q}\left(V-E_{q}\right)$$ (4)

Equation 4 serves as a cornerstone for constructing practical neurodynamic models. Despite its success, the approximate equivalence between Eqs. 3 and 4 and the integration of electrodiffusive dynamics into conductance-based models remain open questions.

In the steady state, we find that for neural systems illustrated in Fig 1, enforcing overall electrical neutrality and replacing Poisson’s equation with Gauss’s law simplifies the PNP problem to a one-dimensional form:


$$\frac{d[q{]}_{i}}{dt}=-\frac{d[q{]}_{o}}{dt}=\frac{S}{v}J_{q}$$ (5)

$$\begin{array}{c} J_{q}=-D_{q}\left(\frac{\partial c_{q}(x)}{\partial x}+\frac{z_{q}F}{RT}c_{q}(x)\frac{\partial \varphi (x)}{\partial x}\right) \end{array}$$ (6)

$$V=\frac{vF}{2SC_{m}}\left(-{\left[A^{-}\right]}_{i}+\sum_{q}z_{q}\left([q{]}_{i}-[q{]}_{o}\right)\right)$$ (7)

where $q=K^{+},Na^{+},{Cl}^{-}$. The concentration of impermeable anions, ${\left[A^{-}\right]}_{i}$, is set to 110 mM in this work [56,70]. The full derivations are detailed in S1 Appendix.

We analyze neural dynamics in a closed system, meaning the total intracellular and extracellular ion concentrations satisfy $[q{]}_{i}+[q{]}_{o}=constant$. Consequently, from Eq. 7, the membrane potential V evolves according to:


$$C_{m}\frac{dV}{dt}=\frac{vF}{S}\sum_{q}z_{q}\frac{d[q{]}_{i}}{dt}=\sum_{q}{z_{q}FJ}_{q}$$ (8)

where $J_{q}$ is given by Eq. 6, which can be rewritten as:


$$\begin{array}{c} \frac{J_{q}}{c_{q}(x)}=\;-\frac{z_{q}FD_{q}}{RT}\frac{\partial \left(\frac{RT}{z_{q}F}\ln\left(c_{q}(x)\right)+\varphi (x)\right)}{\partial x} \end{array}$$

By integrating this equation from 0 to d, we can obtain:


$$\begin{array}{c} J_{q}\int_{0}^{d}\frac{1}{c_{q}(x)}dx=-\frac{z_{q}FD_{q}}{RT}\left(\varphi_{i}-\varphi_{o}-\frac{RT}{z_{q}F}\ln\left(\frac{[q{]}_{o}}{[q{]}_{i}}\right)\right) \end{array}$$ (9)

Combining Eqs. 8 and 9, we obtain:


$$C_{m}\frac{dV}{dt}=-\sum_{q}{\frac{z_{q}^{2}F^{2}P_{q}}{RT}\frac{d}{\int_{0}^{d}\frac{1}{c_{q}(x)}dx}(V-E}_{q})$$ (10)

where $P_{q}=D_{q}/d$ defines the membrane permeability. From Eq. 10, it follows that the membrane conductance per unit area can be expressed as:


$$G_{q}=\frac{z_{q}^{2}F^{2}P_{q}}{RT}\frac{d}{\int_{0}^{d}\frac{1}{c_{q}(x)}dx}$$ (11)

Eq. 11 reveals that membrane conductance $G_{q}$ is directly proportional to both permeability and the harmonic mean of the intramembrane concentration, which is defined as:


$$\overset{―}{c_{q}}=\frac{d}{\int_{0}^{d}\frac{1}{c_{q}(x)}dx}$$ (12)

Given that the intramembrane concentration profile $c_{q}(x)$ at steady state is obtained in the classical GHK approach [5,6], which is:


$$c_{q}(x)=\frac{[q{]}_{o}-[q{]}_{i}}{1-e^{-\frac{z_{q}FV}{RT}}}e^{-\frac{z_{q}FV}{RTd}x}+\frac{[q{]}_{i}-[q{]}_{o}e^{-\frac{z_{q}FV}{RT}}}{1-e^{-\frac{z_{q}FV}{RT}}}$$ (13)

Using Eq. 13, we compute the harmonic mean concentration $\overset{―}{c_{q}}$ as Eq. 2:


$$\overset{―}{c_{q}}=\frac{[q{]}_{i}-[q{]}_{o}e^{-\frac{z_{q}FV}{RT}}}{1-e^{-\frac{z_{q}FV}{RT}}}\frac{V}{V-\frac{RT}{z_{q}F}\ln\left(\frac{[q{]}_{o}}{[q{]}_{i}}\right)}$$

Notably, by combining Eqs 2, 11, and the circuit equation, we obtain the Goldman-Hodgkin-Katz current equation:


$$I_{q}=G_{q}\left(V-E_{q}\right)=\frac{z_{q}^{2}F^{2}P_{q}}{RT}V\frac{[q{]}_{i}-[q{]}_{o}e^{-\frac{z_{q}FV}{RT}}}{1-e^{-\frac{z_{q}FV}{RT}}}$$ (14)

This demonstrates that our approach effectively bridges the gap between traditional cable models and the electrodiffusive framework. The Gauss-Nernst-Planck formulation provides a means to characterize the conductance of rectifying ion channels and seamlessly incorporate electrodiffusive dynamics into neurodynamic models.

### Characterizing channel rectifications

#### Condition for Linear I-V Relation.

Due to electrodiffusive dynamics, single-channel openings may exhibit nonlinear I-V relationships. Many of these behaviors can be explained by the GHK equation, but quantifying the varying conductance remains challenging. Furthermore, it has been unclear how to separate the respective ionic currents when a channel is permeable to multiple ion species, as in ${GABA}_{A}$ and AMPA channels.

Our GNP approach provides a systematic way to address these issues. Consider ${GABA}_{A}$ and AMPA channels as examples. Both channels are permeable to two ion species. Due to electrodiffusive effects, these channels exhibit a linear I-V relationship under the following condition:


$$\frac{P_{q_{2}}}{P_{q_{1}}}=-\frac{{\left[q_{1}\right]}_{i}-{\left[q_{1}\right]}_{o}}{{\left[q_{2}\right]}_{i}-{\left[q_{2}\right]}_{o}}$$ (15)

where $q_{1}$ and $q_{2}$ denote the two permeant ions, and $P_{q_{1}}$ and $P_{q_{2}}$ represent their respective permeabilities within a single open channel.

#### Channel Conductance and Permeabilities to Specific Permeants.

$GABA_{A}$ channels typically exhibit outward rectification. However, when measuring single-channel conductance, the intracellular and extracellular chloride concentrations are often adjusted to eliminate passive rectification. In contrast, AMPA channels generally display an approximately linear I-V relationship under physiological conditions. Once the linear I-V relationships of $GABA_{A}$ and AMPA channels are measured, the single-channel conductance $g_{c,m}$ can be determined from the slope of the I-V curve.

With $g_{c,m}$ known, the single-channel permeabilities can be computed using the following equations:


$$\begin{array}{c} \left\{ \;\;\;\begin{array}{c} P_{q_{1}}=\frac{RTg_{c,m}}{{S_{c}F}^{2}}\frac{{\left[q_{2}\right]}_{i}-{\left[q_{2}\right]}_{o}}{{\left[q_{1}\right]}_{o}{\left[q_{2}\right]}_{i}-{\left[q_{2}\right]}_{o}{\left[q_{1}\right]}_{i}}\mathrm{\backslash vspace}1mm\\ P_{q_{2}}=\frac{RTg_{c,m}}{{S_{c}F}^{2}}\frac{{\left[q_{1}\right]}_{o}-{\left[q_{1}\right]}_{i}}{{\left[q_{1}\right]}_{o}{\left[q_{2}\right]}_{i}-{\left[q_{2}\right]}_{o}{\left[q_{1}\right]}_{i}} \end{array}\;\right.\; \end{array}$$ (16)

where $S_{c}$ represents the cross-sectional area of the channel pore. Experimental studies have rarely reported single-channel permeability data, yet permeability provides a more intrinsic characterization of channel properties compared to conductance, which depends on ion concentrations and membrane potential.

The individual conductance for $q_{1}$ and $q_{2}$ can be further derived as:


$$\begin{array}{c} \left\{ \;\;\;\begin{array}{c} g_{q_{1}}=\frac{S_{c}F^{2}P_{q_{1}}}{RT}\overset{―}{c_{q_{1}}}\mathrm{\backslash vspace}2mm\\ g_{q_{2}}=\frac{S_{c}F^{2}P_{q_{2}}}{RT}\overset{―}{c_{q_{2}}} \end{array}\;\right.\; \end{array}$$ (17)

where $\overset{―}{c_{q_{1}}}$ and $\overset{―}{c_{q_{2}}}$ represent the harmonic mean concentrations of the respective ions.

Notably, the conductance ratio between $q_{1}$ and $q_{2}$ is given by:


$$\frac{g_{q_{1}}}{g_{q_{2}}}=\frac{P_{q_{1}}}{P_{q_{2}}}\frac{\overset{―}{c_{q_{1}}}}{\overset{―}{c_{q_{2}}}}$$ (18)

This result highlights that the conductance ratio does not necessarily equal the permeability ratio $P_{q_{1}}/P_{q_{2}}$, even though previous studies have often assumed them to be identical.

#### Conductance and Reversal Potential of Ion Channels.

For an entire ion channel, the total current can be expressed as:


$$i_{c}=-g_{c}(V-E_{c})$$ (19)

Interestingly, we found that conductance and reversal potential can be expressed in two distinct forms, both of which effectively characterize the channel’s electrical properties. We refer to the first form as the “apparent” conductance and reversal potential, given by Eqs. 20 and 21:


$$g_{c,a}=\frac{{S_{c}F}^{2}}{RT}P_{q_{1}}\overset{―}{c_{C}}$$ (20)

$$E_{c,a}=\frac{RT}{F}\ln\left(\frac{[C{]}_{o}}{[C{]}_{i}}\right)$$ (21)

Here, $q_{1}$ represents an arbitrary permeant ion, and the intracellular and extracellular concentrations $[C{]}_{i}$ and $[C{]}_{o}$ are defined as:


$$\begin{array}{c} \left\{ \;\;\;\begin{array}{c} {\left[C\right]}_{i}={\left[q_{1}\right]}_{i}+\frac{P_{q_{2}}}{P_{q_{1}}}{\left[q_{2}\right]}_{i}\\ {\left[C\right]}_{o}={\left[q_{1}\right]}_{o}+\frac{P_{q_{2}}}{P_{q_{1}}}{\left[q_{2}\right]}_{o} \end{array}\;\right.\; \end{array}$$ (22)

The term $\overset{―}{c_{C}}$ represents the harmonic mean of [C], given by:


$$\overset{―}{c_{C}}=\frac{[C{]}_{i}-[C{]}_{o}e^{-\frac{z_{C}FV}{RT}}}{1-e^{-\frac{z_{C}FV}{RT}}}\frac{V}{V-\frac{RT}{z_{C}F}\ln\left(\frac{[C{]}_{o}}{[C{]}_{i}}\right)}$$ (23)

Here, $z_{C}$ is set to 1 for AMPA channels and −1 for ${GABA}_{A}$ channels.

The apparent conductance $g_{c,a}$ and reversal potential $E_{c,a}$ remain constant under linear I-V conditions (see Eq. 15). However, when channels exhibit rectification, $E_{c,a}$ remains voltage-independent, whereas $g_{c,a}$ varies with membrane potential.

We have also identified another set of conductance and reversal potential, which we refer to as the “latent” conductance and reversal potential:


$$g_{c,l}=g_{q_{1}}+g_{q_{2}}$$ (24)

$$E_{c,l}=\frac{g_{q_{1}}E_{q_{1}}+g_{q_{2}}E_{q_{2}}}{g_{q_{1}}+g_{q_{2}}}$$ (25)

Unlike their apparent counterparts, both $g_{c,l}$ and $E_{c,l}$ are voltage-dependent, even when the I-V relationship is linear (see Fig 3).

If we consider the entire membrane as a single effective “channel,” Eqs. 21 and 25 can be used to determine the resting membrane potential, corresponding to the Goldman-Hodgkin-Katz voltage equation and the chord conductance equation, respectively.

Crucially, apparent and latent conductance and reversal potentials must be carefully matched in pairs to accurately describe channel electrophysiological properties. However, previous studies have frequently mismatched these parameters, potentially leading to inaccurate interpretations and misleading conclusions. Detailed derivations and more generalized forms of Eqs. 15–25 are provided in the S2 Appendix.

### The electrodiffusive neurodynamic model

#### The GNP neurodynamic model.

Although the previous derivations and analyses are based on steady-state conditions, it is reasonable to extend these results to model neural dynamics, as intramembrane electrodiffusion occurs much faster than voltage dynamics (see S3 Appendix Quasi-static Approximation). This allows us to incorporate channel rectifications into practical electrodiffusive neurodynamic models.

Our model includes three types of ion channels: voltage-gated channels, leak channels, and glutamatergic channels. Additionally, to balance passive ion transport and maintain ionic homeostasis, we incorporate active ion transport via the $Na^{+}/K^{+}-ATPase$ pump. These channels and pumps generate four types of ionic currents: voltage-gated current ($I_{qV}$), leak current ($I_{qL}$), glutamatergic current ($I_{qG}$), and pump-induced current ($I_{NaK}$).

Since the total concentration of each ion type remains constant in our isolated system (${\left[q\right]}_{i}+{\left[q\right]}_{o}=constant$), we select extracellular potassium concentration (${\left[K^{+}\right]}_{o}$), intracellular sodium concentration (${\left[{Na}^{+}\right]}_{i}$), and intracellular chloride concentration (${\left[{Cl}^{-}\right]}_{i}$) as independent dynamic variables. The corresponding dynamic equations in our model are given by:


$$\begin{array}{c} \left\{ \;\;\;\begin{array}{c} \frac{d{\left[K^{+}\right]}_{o}}{dt}=\frac{S}{vF}\left(-I_{KV}-I_{KL}-I_{KG}+2I_{NaK}\right)\\ \frac{d{\left[{Na}^{+}\right]}_{i}}{dt}=\frac{S}{vF}\left(I_{NaV}+I_{NaL}+I_{NaG}+3I_{NaK}\right)\mathrm{\backslash vspace}1mm\\ \;\frac{d{\left[{Cl}^{-}\right]}_{i}}{dt}=\frac{S}{vF}I_{ClL} \end{array}\right.\; \end{array}$$ (26)

The ${\left[K^{+}\right]}_{i}$, ${\left[{Na}^{+}\right]}_{o}$, ${\left[{Cl}^{-}\right]}_{o}$, and membrane potential V are determined by Eq. 27:


$$\begin{array}{c} \{\;\;\;\begin{array}{c} {}*20cV=\frac{vF}{2SC_{m}}\left(-{\left[A^{-}\right]}_{i}+\sum_{q}z_{q}\left({\left[q\right]}_{i}-{\left[q\right]}_{o}\right)\right)\\ {\left[q\right]}_{i/o}=\sigma_{q}-{\left[q\right]}_{o/i},\;\;\;\;\;\;\;q=K^{+},Na^{+},Cl^{-} \end{array}\; \end{array}$$ (27)

where $\sigma_{q}$ are constant parameters.

Leak channel currents are modeled using the GHK current equation:


$$I_{qL}=-\frac{F^{2}P_{qL}}{RT}V\frac{[q{]}_{i}-[q{]}_{o}e^{-\frac{z_{q}FV}{RT}}}{1-e^{-\frac{z_{q}FV}{RT}}},\;q=K^{+},Na^{+},Cl^{-}$$ (28)

Similarly, AMPA channels currents are given by:


$$I_{qG}=-p_{o}(tfracF^{2}P_{qG}RTV\frac{[q{]}_{i}-[q{]}_{o}e^{-\frac{FV}{RT}}}{1-e^{-\frac{FV}{RT}}},\;q=K^{+},Na^{+}$$ (29)

The $P_{qL}$ and $P_{qG}$ are averaged membrane permeabilities, which are calculated as:


$$P_{average}={\frac{{N_{c}S}_{c}}{S}P}_{qc}$$ (30)

Here, $P_{qc}$ represents the single-channel permeabilities, estimated using Eq. 16. $N_{c}/S$ denotes the number of channel c per unit area. The values of $P_{qc}$ and $N_{c}/S$ are provided in the Results section, while the corresponding values of $P_{qL}$ and $P_{qG}$ are listed in Tab 1.

The corresponding conductance $G_{qL}$ and $G_{qG}$ is calculated using Eqs. 1 and 2.

To investigate the interaction between passive and active ion transport, the pump-induced current $I_{NaK}$ is either held constant or varies with ion concentrations, as described by Eq. 31 [24,25]:


$$I_{NaK}=-\frac{vF}{S}M_{NaK}\frac{1}{1+e^{\left(\frac{25-{\left[Na^{+}\right]}_{i}}{3}\right)}}\times \frac{1}{1+e^{\left(3.5-{\left[K^{+}\right]}_{o}\right)}}$$ (31)

The voltage-gated currents are modeled as follows:


$$I_{KV}=-G_{KM}n^{4}(V-E_{K}$$ (32)

$$I_{NaV}=-G_{NaM}m^{3}h(V-E_{Na})$$ (33)

$$\frac{dp}{dt}=\alpha_{p}(1-p)-\beta_{q}p,\;p=m,h,n$$ (34)

To model voltage-gated currents, we use a circuit-based approach and adopt the reduced Traub-Miles (RTM) equations for gating kinetics [50], as specialized gating kinetics for electrodiffusive models have not yet been developed. The gating variables m, h, n follow the RTM kinetics:


$$\begin{array}{c} \left\{ \;\;\;\begin{array}{c} \alpha_{m}=\frac{0.32(V+54)}{1-e^{-\frac{V+54}{4}}},\;\beta_{m}=\frac{0.28(V+27)}{e^{\frac{V+27}{5}}-1}\\ \alpha_{h}=0.128e^{-\frac{V+50}{18}},\;\beta_{h}=\frac{4}{{1+e}^{-\frac{V+27}{5}}}\;\\ \alpha_{n}=\frac{0.032(V+52)}{1-e^{-\frac{V+52}{5}}},\;\beta_{n}=0.5e^{-\frac{V+57}{40}} \end{array}\right.\; \end{array}$$ (35)

#### Concentration-Fixed GNP (fGNP) Model.

Ion concentrations dynamically change during neural activity in this GNP neurodynamic model. However, for short-term neural activity, ion concentration changes may be negligible. In such cases, it is practical to fix ion concentrations and model neural activity using the following dynamic equation:


$$\frac{dV}{dt}=I_{V}+I_{G}+I_{L}+I_{NaK}$$ (36)

where the total voltage-gated current $I_{V}$, glutamatergic current $I_{G}$, and leak current $I_{L}$ are defined as:


$$\begin{array}{c} \left\{ \;\;\;\begin{array}{c} I_{V}=-G_{NaM}m^{3}h\left(V-E_{Na}\right)-G_{KM}n^{4}\left(V-E_{K}\right)\\ \;I_{G}=-p_{o}(t)G_{glu}\left(V-E_{glu}\right)\;\\ \;I_{L}=-G_{L,r}(V-E_{L.r}) \end{array}\right.\; \end{array}$$ (37)

Here, $G_{NaM}$, $G_{KM}$, m, h, n share the same values and dynamic equations as described in Eqs. 32–35. Glutamate channels are assumed to exhibit a linear I-V relationship (see Eq. 15), with $G_{glu}$ and $E_{glu}$ calculated using Eqs. 20–23 (see Tab. 1). Leak channels, however, are assumed to exhibit rectifications due to the significantly higher membrane permeability to potassium than sodium. The latent voltage-dependent conductance $G_{L,r}$ and reversal potential $E_{L,r}$ are expressed as


$$\begin{array}{c} \left\{ \;\;\;\begin{array}{c} G_{L,l}=G_{KL}+G_{NaL}+G_{ClL}\\ E_{L,l}=\frac{G_{KL}E_{K}+G_{NaL}E_{Na}+G_{ClL}E_{Cl}}{G_{K}+G_{Na}+G_{Cl}} \end{array}\right.\; \end{array}$$ (38)

The specific leak conductance for potassium, sodium, and chloride is given by:


$$G_{qL}=\frac{z_{q}^{2}F^{2}P_{qL}\overset{―}{c_{q}}}{RT}$$ (39)

We define the total electrodiffusive current as the sum of voltage-gated, glutamatergic, and leak currents:


$$I_{diff}=I_{V}+I_{G}+I_{L}$$ (40)

In Eq. 36, the active transport current $I_{NaK}$ is set as a constant. The model described by Eqs. 36–39 is referred to as the ion concentration-fixed GNP (fGNP) model.

The fGNP model closely resembles traditional conductance-based models, with the primary distinction being the introduction of rectifying leak channels (Eq. 39). To illustrate the impact of this modification, we also construct a classical Hodgkin-Huxley (cHH) model incorporating a linear leak current:


$$I_{LH}=-G_{LH}(V-E_{LH})$$ (41)

where $G_{LH}$ and $E_{LH}$ are constants, matched to $G_{L,l}$ and $E_{L,l}$ in the fGNP model at steady state. The currents $I_{V}$ and $I_{G}$ are identical in both the fGNP and cHH models.

#### GNP model with modified voltage-gated currents.

In both the fGNP and GNP models, electrodiffusive effects within voltage-gated channels are neglected, an assumption valid only when ion concentration changes are minimal. However, when ion concentration dynamics become significant, this simplification becomes insufficient, as traditional RTM equations overlook the concentration dependence of conductance. To address this, we model voltage-gated currents using the GHK current equation:


$$\begin{array}{c} \left\{ \;\;\;\begin{array}{c} I_{KV}^{\prime }=-\frac{F^{2}P_{KV}}{RT}\;n^{4}V\frac{{\left[K^{+}\right]}_{i}-{\left[K^{+}\right]}_{o}e^{-\frac{FV}{RT}}}{1-e^{-\frac{FV}{RT}}}\\ I_{NaV}^{\prime }=-\frac{F^{2}P_{NaV}}{RT}\;m^{3}hV\frac{{\left[{Na}^{+}\right]}_{i}-{\left[{Na}^{+}\right]}_{o}e^{-\frac{FV}{RT}}}{1-e^{-\frac{FV}{RT}}} \end{array}\right.\; \end{array}$$ (42)

The gating variables m, h, and n follow the same dynamics as in Eq. 35. Assuming the RTM equations accurately capture voltage-gated current rectification under steady-state ion concentrations, we introduce a normalization factor $f_{q}(V)$ such that $I_{qV}^{\prime }/f_{q}(V)=I_{qV}$ (at steady state). The function $f_{q}(V)$ is fitted using a cubic equation (Fig 8a, 8b, lower panels):


$$f_{q}=N_{q3}V^{3}+N_{q2}V^{2}+N_{q1}V+N_{q0}$$ (43)

Using $f_{q}(V)$, the dynamic equations for extracellular potassium and intracellular sodium concentrations are:


$$\left\{ \begin{array}{c} \frac{d{\left[K^{+}\right]}_{o}}{dt}=\frac{S}{vF}\left(-\frac{I_{KV}^{\prime }}{f_{K}(V)}-I_{KL}-I_{KG}+2I_{NaK}\right)\\ \frac{d{\left[{Na}^{+}\right]}_{i}}{dt}=\frac{S}{vF}\left(\frac{I_{NaV}^{\prime }}{f_{Na}(V)}+I_{NaL}+I_{NaG}+3I_{NaK}\right) \end{array}\;\right.\;$$ (44)

The model incorporating Eq. 44 is referred to as the vGNP model. The equivalent maximum conductance for voltage-gated currents in the vGNP model is given by:


$$G_{qM}^{\prime }=\frac{F^{2}P_{qV}}{RTf_{q}(V)}\overset{―}{c_{q}}$$ (45)

To analyze how variations in $G_{KM}^{\prime }$ and $G_{NaM}^{\prime }$ influence electrodiffusive currents, we compute the differences in ionic currents between the vGNP and GNP models:


$$\begin{array}{c} \left\{ \;\;\;\begin{array}{c} \;dI_{K^{+}}=-F\left(\frac{d{\left[K^{+}\right]}_{o}^{vGNP}}{dt}-\frac{d{\left[K^{+}\right]}_{o}^{GNP}}{dt}\right)+(2I_{NaK}^{vGNP}-2I_{NaK}^{GNP})\\ {dI}_{Na^{+}}=F\left(\frac{d{\left[{Na}^{+}\right]}_{i}^{vGNP}}{dt}-\frac{d{\left[{Na}^{+}\right]}_{i}^{GNP}}{dt}\right)-(3I_{NaK}^{vGNP}-3I_{NaK}^{GNP})\\ \;dI_{Cl^{-}}=-F\left(\frac{d{\left[{Cl}^{-}\right]}_{i}^{vGNP}}{dt}-\frac{d{\left[{Cl}^{-}\right]}_{i}^{GNP}}{dt}\right) \end{array}\right.\; \end{array}$$ (46)

The $dI_{q}$ calculated through simulated data exhibits dramatic oscillation, and it hard to identify its changing trend. We apply a sliding window with an 8-second width and a 4-second step to smooth oscillations in $dI_{q}$, as shown in Fig 8h. The total difference in electrodiffusive currents, $dI_{diff}$, is the sum of $dI_{K^{+}}$, $dI_{{Na}^{+}}$ and $dI_{Cl^{-}}$, depicted in Fig 8i.

To separately analyze the effects of $G_{KM}^{\prime }$ and $G_{NaM}^{\prime }$ on neural dynamics, we introduce two specialized models: kvGNP and navGNP.

kvGNP Model: Only the voltage-gated potassium current follows the GHK equation, while sodium currents are modeled using the RTM framework:


$$\begin{array}{c} \left\{ \;\;\;\;\begin{array}{c} \frac{d{\left[K^{+}\right]}_{o}}{dt}=\frac{S}{vF}\left(-\frac{I_{KV}^{\prime }}{f_{K}(V)}-I_{KL}-I_{KG}+2I_{NaK}\right)\\ \frac{d{\left[{Na}^{+}\right]}_{i}}{dt}=\frac{S}{vF}\left(I_{NaV}+I_{NaL}+I_{NaG}+3I_{NaK}\right) \end{array}\;\right.\; \end{array}$$ (47)

navGNP Model: Here, the sodium current follows the GHK equation, while potassium currents adhere to the RTM model


$$\begin{array}{c} \left\{ \;\;\;\begin{array}{c} \;\frac{d{\left[K^{+}\right]}_{o}}{dt}=\frac{S}{vF}\left(-I_{KV}-I_{KL}-I_{KG}+2I_{NaK}\right)\\ \frac{d{\left[{Na}^{+}\right]}_{i}}{dt}=\frac{S}{vF}\left(\frac{I_{NaV}^{\prime }}{f_{Na}(V)}+I_{NaL}+I_{NaG}+3I_{NaK}\right) \end{array}\;\right.\; \end{array}$$ (48)

In both models, $I_{qV}$ follows the RTM model (Eq. 35). The dynamics of kvGNP and navGNP are illustrated in S3 Fig.

#### Classical Hodgkin-Huxley Type Model with Ion Concentration Dynamics.

The conductance-based models are frequently utilized in investigating the pathological neural activities like seizures and CSD [23–25,54]. To compare it between our electrodiffusive GNP model, we construct a conductance-based model, denoted as iHH model.

The main kinetic equations for membrane potential and ion concentration within the iHH model are presented as:


$$\frac{dV}{dt}=I_{V}+I_{G}+I_{L}+I_{NaK}$$ (49)

$$\frac{d{\left[K^{+}\right]}_{o}}{dt}=\frac{S}{vF}\left(-I_{KV}-I_{KL}-I_{KG}+2I_{NaK}\right)$$ (50)

$$\frac{d{\left[{Na}^{+}\right]}_{i}}{dt}=\frac{S}{vF}\left(I_{NaV}+I_{NaL}+I_{NaG}+3I_{NaK}\right)$$ (51)

$$\frac{d{\left[{Cl}^{-}\right]}_{i}}{dt}=\frac{S}{vF}I_{ClL}$$ (52)

Where the $I_{NaK}$, $I_{KV}$, $I_{NaV}$ are the same as those in Eqs. 31–33. The currents through leaky and glutamatergic channels are presented as:


$$I_{qL}=-G_{qL,iHH}\left(V-E_{q}\right),\;q=K^{+},Na^{+},Cl^{-}$$ (53)

$$I_{qG}=-p_{o}(t)G_{qG,iHH}\left(V-E_{q}\right),\;q=K^{+},Na^{+}$$ (54)

The $G_{qL,iHH}$ and $G_{qG,iHH}$ are set to be constant, same to those in GNP model at steady state (see Table 1).

**Table 1:** Units and description of the parameters used in the neurodynamic models.

	Value	Description
$P_{KL}$	$2.00\times 10^{-8}\;m/s$	Average leaky permeability for potassium ions
$P_{NaL}$	$1.00\times 10^{-9}\;m/s$	Average leaky permeability for sodium ions
$P_{ClL}$	$4.00\times 10^{-9}\;m/s$	Average leaky permeability for chloride ions
$P_{KG}$	$2.82\times 10^{-8}\;m/s$	Average glutamate permeability for potassium ions
$P_{NaG}$	$2.45\times 10^{-9}\;m/s$	Average glutamate permeability for sodium ions
$P_{KV}$	$1.70\times 10^{-6}\;m/s$	Average voltage-gated permeability for potassium ions
$P_{NaV}$	$1.47\times 10^{-6}m/s$	Average voltage-gated permeability for sodium ions
$G_{KM}$	$20\;mS/{cm}^{2}$	Maximum voltage-gated potassium conductance
$G_{NaM}$	$30\;mS/{cm}^{2}$	Maximum voltage-gated sodium conductance
$G_{glu}$	$0.12\;mS/cm^{2}$	Maximum glutamatergic conductance
$G_{LH}$	$0.17\;mS/cm^{2}$	Leaky conductance in cHH model
$G_{KL,iHH}$	$0.10\;mS/cm^{2}$	Leaky potassium conductance in iHH model
$G_{NaL,iHH}$	$0.030\;mS/cm^{2}$	Leaky sodium conductance in iHH model
$G_{ClL,iHH}$	$0.042\;mS/cm^{2}$	Leaky chloride conductance in iHH model
$G_{KG,iHH}$	$0.12\;mS/cm^{2}$	Glutamatergic potassium conductance in iHH model
$G_{NaG,iHH}$	$0.03\;mS/cm^{2}$	Glutamatergic sodium conductance in iHH model
$E_{glu}$	0 mV	Reversal potential for AMPA channels
$E_{LH}$	−61.52 mV	Leaky reversal potential in cHH model
$M_{NaK}$	0.3 or 0.7 mM/s	Maximum Na-K pump strength
$I_{NaK}$	$-1.16\;\mu A/{cm}^{2}$	Constant pump-induced current
S/v	$4\times 10^{5}\;m^{-1}$	Ratio of surface area to volume
T	309.15 K	Temperature
${\left[A^{-}\right]}_{i}$	110 mM	Concentration of intracellular impermeable anions
$C_{m}$	$1\;\mu F/{cm}^{2}$	Membrane capacitance
${\left[K^{+}\right]}_{o,t=0}$	3.17 mM	Extracellular potassium concentration at steady state
${\left[{Na}^{+}\right]}_{i,t=0}$	23.58 mM	Intracellular sodium concentration at steady state
${\left[{Cl}^{-}\right]}_{i,t=0}$	10.41 mM	Intracellular chloride concentration at steady state
$V_{t=0}$	−68.17 mV	Membrane potential at steady state
$\sigma_{K^{+}}$	100 mM	Conservation constant for potassium ions
$\sigma_{{Na}^{+}}$	155 mM	Conservation constant for sodium ions
$\sigma_{{Cl}^{-}}$	145 mM	Conservation constant for chloride ions

## Supporting information

S1 Appendix Detailed derivations for establishing Gauss-Nernst-Planck Model. (DOCX)

S2 Appendix Characterizing channel properties with GNP approach. (DOCX)

S3 Appendix Quasi-static Approximation. (DOCX)

S1 FigApparent and latent conductance and reversal potentials reproduce the I-V curves of Ion channels.(a) I-V curve for ${GABA}_{A}$ channel. At V=−62.34 mV, the current through the ${GABA}_{A}$ channel is 0 pA, marked by the blue circle. The apparent and latent reversal potentials are identical and equal to V=−62.34 mV, as shown at the inset. However, the apparent and latent conductance, $G_{r,a}$ and $G_{r,c}$, differs at this voltage, represented by the slopes of the black solid and dashed lines, respectively. The red curve depicts the current through the ${GABA}_{A}$ channel. (b) At V=−5.00 mV, both the apparent and latent conductance increases. The apparent reversal potential remains constant, while the latent reversal potential (dashed black line) shifts downward, as shown at the inset. (c) The linear I-V relationship of an individual calcium-impermeable AMPA channel (red curve) can be characterized by the latent conductance and reversal potential, demonstrated here by three cases (black dashed lines) at V=−80 mV, V=0 mV and V=80 mV. (d) An individual GluR2-lacking AMPA channel, permeable to calcium ions, exhibits inward rectification (red curve). The apparent conductance and reversal potential are not applicable in this case, but the latent conductance and reversal potential successfully reproduce the I-V curve, as illustrated by three cases (black dashed lines) at V=−80 mV, V=0 mV and V=80 mV.(TIF)

S2 FigValues of Tim across different locations and voltages.(a-d): For $z_{q}=+1$. (e-h): For $z_{q}=-1$. (i-l): For $z_{q}=+2$. The function Tim reaches its maximum value of approximately 0.125 when V=0 mV and x=d/2.(TIF)

S3 FigNeural dynamics in kvGNP and navGNP models in response to glutamate stimuli.(a) Neural discharge events in kvGNP (upper panel) and navGNP (lower panel) models. The glutamate stimuli are identical to those used in Fig 8d ($p_{o}=0.17$). Both models exhibit tonic-spiking rather than entering a DB state. (b, c) Changes in extracellular potassium (b) and intracellular sodium (c) concentrations for four different models. Insets: zoomed in version of concentration changes between 40 s and 70 s. (d) Timing differences in neural discharge events in kvGNP (middle panel), navGNP (lower panel) models relative to the GNP model, illustrating the lag and lead in action potential timing. Upper panel: Timing of discharge events in the GNP model. (e, f) Differences in electrodiffusive potassium, sodium, and chloride currents in kvGNP (e) and navGNP (f) models, relative to the GNP model. Note that in the kvGNP model, the potassium current can be less inhibitory than in the GNP model, while in the navGNP model, the sodium current may be less excitatory during certain periods.(TIF)
