# Supplementary material for: Investigate channel rectifications and neural dynamics by an electrodiffusive Gauss-Nernst-Planck approach
Source: PLoS Comput Biol. 2025 Jun 30;21(6):e1012883. doi: 10.1371/journal.pcbi.1012883 (PMC12208492; doi:10.1371/journal.pcbi.1012883)
Supplement: S1 Appendix — (DOCX) [file pcbi.1012883.s001.docx]

Applying the overall electroneutrality assumption and Gauss’s law significantly simplifies the Poisson-Nernst-Planck problem in neural systems. Below, we outline how these conditions simplify the PNP equation (Eq. 3).

The total charge in the intracellular and extracellular spaces is given by:

$Q_{i}=vF\sum_{q} z_{q}\left[ q \right]_{i}$ (S1)

$Q_{o}=vF\sum_{q} z_{q}\left[ q \right]_{o}$ (S2)

where $q$ denotes a specific ion species with valence $z_{q}$​ and concentrations $\left[ q \right]_{i}$ and $\left[ q \right]_{o}$ in the intracellular and extracellular spaces, respectively. $F$ is Faraday’s constant, and $v$ represents volume, which is assumed to be equal in both compartments.

To maintain overall electroneutrality, we require:

$$Q_{i}+Q_{o}=0 C$$

According to Gauss’s law, the electric field in the intramembrane space remains constant and is given by:

$E=\frac{Q}{\epsilon_{0}\epsilon_{r}S}$ (S3)

where $Q=|Q_{i}-Q_{o}|/2$, $S$ is the surface area of the neural membrane within the intramembrane space (e.g., surface $S2$​ in Fig. 1a, 1b), and $\epsilon_{0}\epsilon_{r}$​ represents membrane permittivity. This formulation aligns with the constant-field theory [5, 6]. Crucially, using Gauss’s law allows us to avoid solving the Poisson equation directly. Instead, we can express the membrane potential as a function of ion concentrations:

$V=-E*d=\frac{vF}{{2C}_{m}S}\sum_{q} z_{q}\left( \left[ q \right]_{i}-\left[ q \right]_{o} \right)$ (S4)

where $d$ is the intramembrane distance, and $C_{m}=\epsilon_{0}\epsilon_{r}/d$ represents the membrane capacitance per unit area.

We assume the neural membrane is permeable to potassium, sodium, and chloride ions. Under physiological conditions, their typical intra- and extracellular concentrations are:

$$\left[ K^{+} \right]_{i}=96 mM, \left[ K^{+} \right]_{o}=4 mM$$

$$\left[ {Na}^{+} \right]_{i}=20 mM, \left[ {Na}^{+} \right]_{o}=135 mM$$

$$\left[ {Cl}^{-} \right]_{i}=6 mM, \left[ {Cl}^{-} \right]_{o}=139 mM$$

From these values, the intracellular and extracellular charge densities are:

$$c_{i}=\left[ K^{+} \right]_{i}+\left[ {Na}^{+} \right]_{i}-\left[ {Cl}^{-} \right]_{i}=110 mM$$

$$c_{o}=\left[ K^{+} \right]_{o}+\left[ {Na}^{+} \right]_{o}-\left[ {Cl}^{-} \right]_{o}=0 mM$$

Substituting these into Eq. S4 results in an unrealistically high membrane potential exceeding ${10}^{6} mV$, which is not observed in biological systems. This discrepancy highlights the necessity of including impermeable intracellular anions ($A^{-}$) to balance the charge distribution. These anions are abundant in the intracellular space but scarce extracellularly. Thus, we introduce:

$$\left[ A^{-} \right]_{i}=110 mM, \left[ A^{-} \right]_{o}=0 mM$$

to maintain charge neutrality and produce a physiologically realistic membrane potential.

Notably, Eq. S4 establishes a direct relationship between voltage dynamics and ion concentration dynamics:

$C_{m}\frac{dV}{dt}=\frac{vF}{S}\sum_{q} z_{q}\left( \frac{d\left[ q \right]_{i}}{dt}-\frac{d\left[ q \right]_{o}}{dt} \right)$ (S5)

As illustrated in Fig. 1c, we assume ions are uniformly distributed within the intra- and extracellular spaces while continuously varying within the intramembrane space. At equilibrium, the diffusive flux $J_{qdiff}$​ of ion $q$ must remain constant across the neural membrane, as defined in Eq. S6:

$J_{\mathrm{qdiff}}(x,t)=\left\{ \begin{aligned} 0, x<0 \\ J_{q}, 0\leq x\leq d \\ 0, x>d \end{aligned} \right.$ (S6)

Under these conditions, the dynamic equations for $\left[ q \right]_{i}$ and $\left[ q \right]_{o}$ are:

$\frac{d\left[ q \right]_{i}}{dt}=\frac{S}{v}J_{qdiff}\left( d,t \right)=\frac{S}{v}J_{q}$ (S7)

$\frac{d\left[ q \right]_{o}}{dt}=-\frac{S}{v}J_{qdiff}\left( 0,t \right)=-\frac{S}{v}J_{q}$ (S8)

where $J_{q}$​ is given by the Nernst-Planck equation (Eq. 6 in Methods).

These derivations, summarized in Eqs. 5–6 in Methods, form the foundation for constructing our electrodiffusive neurodynamic model.
