# Supplementary material for: Investigate channel rectifications and neural dynamics by an electrodiffusive Gauss-Nernst-Planck approach
Source: PLoS Comput Biol. 2025 Jun 30;21(6):e1012883. doi: 10.1371/journal.pcbi.1012883 (PMC12208492; doi:10.1371/journal.pcbi.1012883)
Supplement: S2 Appendix — (DOCX) [file pcbi.1012883.s002.docx]

In experimental studies, the measured channel current ($i_{c}$) and conductance ($g_{c}$) are related to their respective current ($I_{c}$​, Eq. 18) and conductance ($G_{c}$​, Eq. 1) per unit area as follows:

$i_{c}=S_{c}I_{c}, g_{c}=S_{c}G_{c}$ (S9)

where $S_{c}$ represents the cross-sectional area of the channel's pore domain.

For ion channels permeable to multiple ion types, the total channel current can be expressed as:

$i_{c}=\sum_{q} z_{q}S_{c}FJ_{q}=-\frac{{S_{c}F}^{2}}{RT}\sum_{q} P_{q}V\frac{{[q]}_{i}-{[q]}_{o}e^{-\frac{z_{q}FV}{RT}}}{1-e^{-\frac{z_{q}FV}{RT}}}$ (S10)

For channels permeable only to monovalent ions, Eq. S10 simplifies to:

$i_{c}=-\frac{{S_{c}F}^{2}}{RT}P_{C}V\frac{\left[ C \right]_{i}-\left[ C \right]_{o}e^{-\frac{FV}{RT}}}{1-e^{-\frac{FV}{RT}}}$ (S11)

where $P_{C}{[C]}_{i}$​ and $P_{C}{[C]}_{o}$​ are constants. The value of $P_{C}$​ is set equal to one of the permeant ion permeabilities (e.g., $P_{C}=P_{q_{1}}$​​). The equivalent concentrations ${[C]}_{i}$ and ${[C]}_{o}$​ are defined as:

${[C]}_{i}=\sum_{q^{+}} \frac{P_{q^{+}}}{P_{C}}{[q^{+}]}_{i}+\sum_{q^{-}} \frac{P_{q^{-}}}{P_{C}}{[q^{-}]}_{o}$ (S12)

${[C]}_{o}=\sum_{q^{+}} \frac{P_{q^{+}}}{P_{C}}{[q^{+}]}_{o}+\sum_{q^{-}} \frac{P_{q^{-}}}{P_{C}}{[q^{-}]}_{i}$ (S13)

where $q^{+}$ and $q^{-}$ represent monovalent cations and anions, respectively.

When ${[C]}_{i}={[C]}_{o}$, the channel exhibits a linear I-V relationship, and Eq. S11 reduces to:

$i_{c}=-\frac{{S_{c}F}^{2}}{RT}P_{C}[C]V$ (S14)

where $\left[ C \right]=\left[ C \right]_{i}$. The apparent channel conductance, corresponding to the slope of the linear I-V curve, is given by:

$g_{c,a}=\frac{{S_{c}F}^{2}}{RT}P_{C}[C]$ (S15)

Equations S11–S15 provide a framework for estimating ion channel permeabilities from experimental data.

Consider a channel with two types of permeants, $q_{1}$ and $q_{2}$​, both with the same valence. Setting $P_{C}=P_{q_{1}}$​​, the ion concentrations and permeability ratio must satisfy Eq. S16 to achieve ${[C]}_{i}={[C]}_{o}$​ in order to maintain a linear I-V relationship:

$\left[ q_{1} \right]_{i}+\frac{P_{q_{2}}}{P_{q_{1}}}\left[ q_{2} \right]_{i}=\left[ q_{1} \right]_{o}+\frac{P_{q_{2}}}{P_{q_{1}}}\left[ q_{2} \right]_{o}$ (S16)

Solving for the permeability ratio:

$$\frac{P_{q_{2}}}{P_{q_{1}}}=-\frac{\left[ q_{1} \right]_{i}-\left[ q_{1} \right]_{o}}{\left[ q_{2} \right]_{i}-\left[ q_{2} \right]_{o}}$$

The total current then becomes:

$i_{c}=-\frac{S_{c}F^{2}}{RT}P_{q_{1}}\left( \frac{\left[ q_{1} \right]_{o}\left[ q_{2} \right]_{i}-\left[ q_{2} \right]_{o}\left[ q_{1} \right]_{i}}{\left[ q_{2} \right]_{i}-\left[ q_{2} \right]_{o}} \right)V$ (S17)

If ion concentrations $\left[ q \right]$, single-channel conductance $g_{c,m}$​, and pore diameter $S_{c}$​ are known experimentally, the permeabilities $P_{q_{1}}$ and $P_{q_{2}}$​​ can be computed using Eq. 16:

$$P_{q_{1}}=\frac{RTg_{c,m}}{{S_{c}F}^{2}}\frac{\left[ q_{2} \right]_{i}-\left[ q_{2} \right]_{o}}{\left[ q_{1} \right]_{o}\left[ q_{2} \right]_{i}-\left[ q_{2} \right]_{o}\left[ q_{1} \right]_{i}}$$

$$P_{q_{2}}=\frac{RTg_{c,m}}{{S_{c}F}^{2}}\frac{\left[ q_{1} \right]_{o}-\left[ q_{1} \right]_{i}}{\left[ q_{1} \right]_{o}\left[ q_{2} \right]_{i}-\left[ q_{2} \right]_{o}\left[ q_{1} \right]_{i}}$$

Once permeabilities are determined, the conductance for $q_{1}$ and $q_{2}$​ can be calculated as Eq. 17:

$$g_{q_{1}}=\frac{S_{c}F^{2}P_{q_{1}}}{RT}\bar{c_{q_{1}}}$$

$$g_{q_{2}}=\frac{S_{c}F^{2}P_{q_{2}}}{RT}\bar{c_{q_{2}}}$$

When ${[C]}_{i}\neq{[C]}_{o}$​, the channel exhibits rectification, and the total current is given by:

$i_{c}=-g_{c,a}\left( V-E_{c,a} \right)$ (S18)

where the apparent conductance is:

$g_{c,a}=\frac{{S_{c}F}^{2}}{RT}P_{C}\bar{c_{C}}$ (S19)

and the apparent reversal potential is:

$E_{c,a}=\frac{RT}{F}ln(\frac{\left[ C \right]_{o}}{\left[ C \right]_{i}})$ (S20)

Interestingly, Eq. S18 can be rewritten in a circuit-like form:

$i_{c}=-\sum_{q} g_{q}\left( V-E_{q} \right)=-g_{c,l}\left( V-E_{c,l} \right)$ (S21)

where we define the latent conductance and reversal potential as:

$g_{c,l}=\sum_{q} g_{q}$ (S22)

$E_{c,l}=\frac{\sum_{q} g_{q}E_{q}}{\sum_{q} g_{q}}$ (S23)

Both $(g_{c,a},E_{c,a})$, and $(g_{c,l},E_{c,l})$ can reproduce the channel’s I-V curve. However, while $g_{c,a}$ and $E_{c,a}$​ are only valid for channels with equivalent permeants, $g_{c,l}$ and $E_{c,l}$ have no such limitation. Further details on apparent and latent conductance and reversal potentials are provided in Supporting Information Figure 1.
