# Supplementary material for: Investigate channel rectifications and neural dynamics by an electrodiffusive Gauss-Nernst-Planck approach
Source: PLoS Comput Biol. 2025 Jun 30;21(6):e1012883. doi: 10.1371/journal.pcbi.1012883 (PMC12208492; doi:10.1371/journal.pcbi.1012883)
Supplement: S3 Appendix — (DOCX) [file pcbi.1012883.s003.docx]

To investigate the impact of electrodiffusion on neural dynamics during transient neural activity, we assume that electrodiffusive dynamics within channel pores occur much faster than voltage dynamics. Consequently, these dynamics can be approximated as a series of "quasi-static" states during neural spikes. To validate this assumption, we estimate the characteristic timescale for the intramembrane concentration profile to transition from $c_{q}(x,V_{0})$ to $c_{q}(x,V_{0}+\Delta V)$ in response to a small membrane potential perturbation $\Delta V$.

In general, the temporal evolution of the concentration profile within an ion channel follows:

$\frac{dc_{q}(x,V)}{dt}=-\frac{\partial J_{diff}\left( x,V \right)}{\partial x}$ (S24)

Assuming a steady state at $V=V_{0}$ ​, we obtain:

$\frac{dc_{q}(x,V_{0})}{dt}=-\frac{\partial J_{diff}\left( x,V_{0} \right)}{\partial x}=0$ (S25)

which implies to:

$D_{q}\left( \frac{\partial^{2}c_{q}\left( x,V_{0} \right)}{\partial x^{2}}+\frac{z_{q}FV}{RTd}\frac{\partial c_{q}\left( x,V_{0} \right)}{\partial x} \right)=0$ (S26)

If the membrane potential shifts to $V_{0}+\Delta V$ while the concentration profile remains temporarily at $c_{q}(x,V_{0})$, the rate of change in concentration, $dc_{q}(x,V)/dt$, can be approximated as:

$\frac{dc_{q}(x,V)}{dt}\approx\frac{z_{q}FP_{q}}{RT}\Delta V\frac{\partial c_{q}\left( x,V_{0} \right)}{\partial x}$ (S27)

Meanwhile, the new steady-state profile $c_{q}\left( x,V_{0}+\Delta V \right)$ can be expressed as:

$c_{q}\left( x,V_{0}+\Delta V \right)\approx c_{q}\left( x,V_{0} \right)+\frac{\partial c_{q}\left( x,V_{0} \right)}{\partial V}\Delta V$ (S28)

The characteristic time required for the concentration profile to shift from $c_{q}\left( x,V_{0} \right)$ to $c_{q}\left( x,V_{0}+\Delta V \right)$, denoted as $\Delta t$, can be estimated as:

$\Delta t=\frac{c_{q}\left( x,V_{0}+\Delta V \right)-c_{q}\left( x,V_{0} \right)}{\frac{dc_{q}\left( x,V \right)}{dt}}=\frac{\frac{\partial c_{q}\left( x,V_{0} \right)}{\partial V}\Delta V}{\frac{z_{q}FP_{q}}{RT}\Delta V\frac{\partial c_{q}(x,V_{0})}{\partial x}}$ (S29)

Reorganizing Eq. S29 gives:

$\Delta t=\frac{d}{P_{q}}Tim$ (S30)

where $Tim$ is expressed as:

$Tim=\frac{\xi}{e^{-\frac{\xi x}{d}}}\times\frac{\left( \left( \left( 1-\frac{x}{d} \right)e^{-\xi}+\frac{x}{d} \right)e^{-\frac{\xi x}{d}}-e^{-\xi} \right)}{1-e^{-\xi}}$ (S31)

with $\xi=\frac{z_{q}FV_{0}}{RT}$​​. The function $Tim$ reaches its maximum value at $x=d/2$ when $V_{0}$​ approaches $0 mV$. Notably, for $z_{q}=1$, the maximum $Tim$ is approximately 0.125 (see Supporting Information Figure 2).

According to Eq. S30, $\Delta t$ is directly proportional to the membrane thickness $d$ and inversely proportional to permeability $P_{q}$​​. As estimated in the main text, the permeabilities of ${GABA}_{A}$ and $AMPA$ channels are on the order of ${10}^{-2} m/s$. Given a membrane thickness of approximately $10 nm$, $\Delta t$ is estimated to be less than ${10}^{-6} s$. Since typical membrane potential changes occur on a timescale of ${10}^{-3} s$, this $\Delta t$ is negligible. This analysis supports the assumption that electrodiffusive dynamics can be approximated as a series of quasi-static states during dynamic neural events, justifying the application of steady-state equations (Eqs. 5–14) from our GNP model to describe neural dynamics.
