# Supplementary figures and images for: Investigate channel rectifications and neural dynamics by an electrodiffusive Gauss-Nernst-Planck approach
Source: PLoS Comput Biol. 2025 Jun 30;21(6):e1012883. doi: 10.1371/journal.pcbi.1012883 (PMC12208492; doi:10.1371/journal.pcbi.1012883)

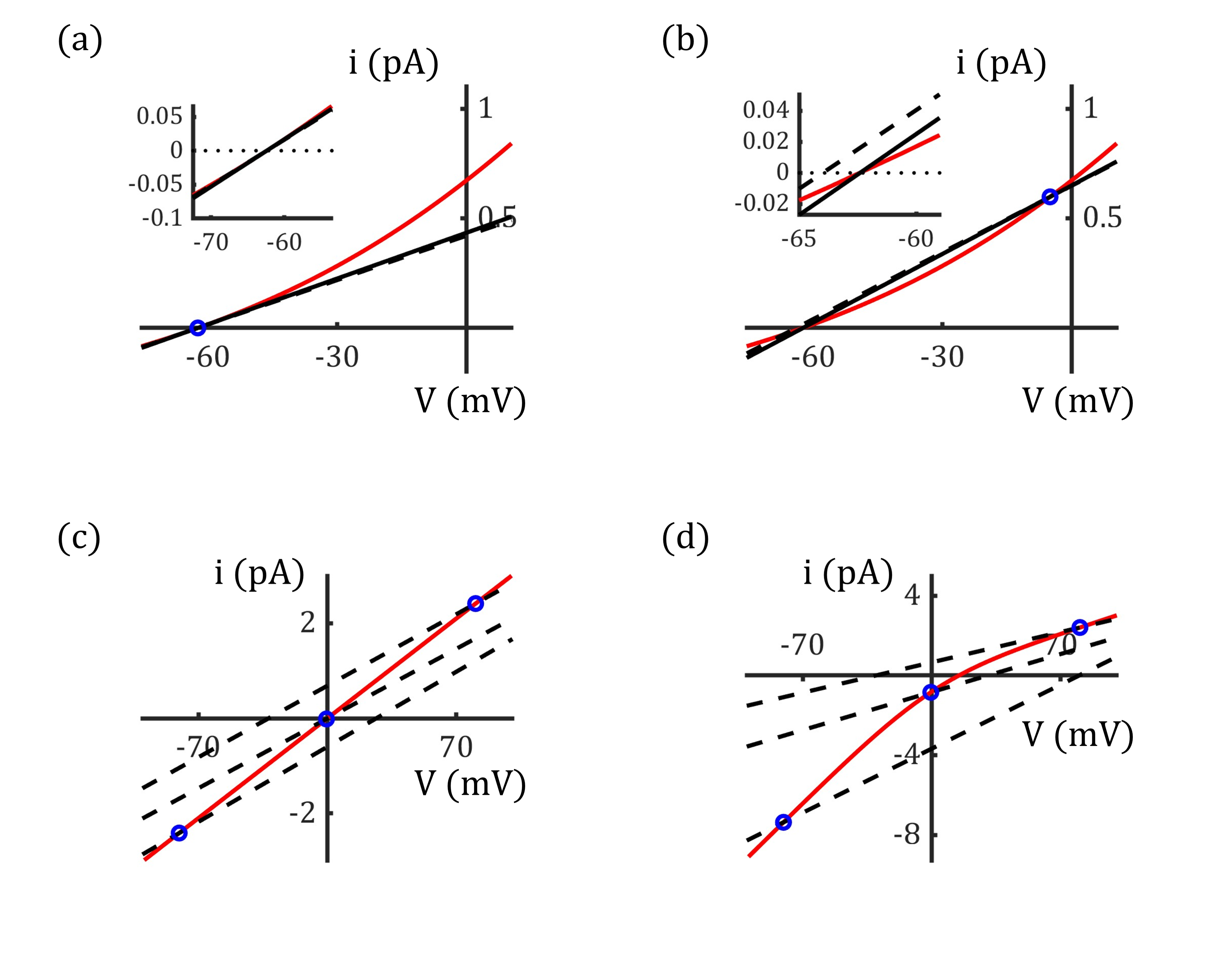

Supplement: S1 Fig — (a) I-V curve for GABAA channel. At V=−62.34 mV, the current through the GABAA channel is 0 pA, marked by the blue circle. The apparent and latent reversal potentials are identical and equal to V=−62.34 mV, as shown at the inset. However, the apparent and latent conductance, Gr,a and Gr,c, differs at this voltage, represented by the slopes of the black solid and dashed lines, respectively. The red curve depicts the current through the GABAA channel. (b) At V=−5.00 mV, both the apparent and latent conductance increases. The apparent reversal potential remains constant, while the latent reversal potential (dashed black line) shifts downward, as shown at the inset. (c) The linear I-V relationship of an individual calcium-impermeable AMPA channel (red curve) can be characterized by the latent conductance and reversal potential, demonstrated here by three cases (black dashed lines) at V=−80 mV, V=0 mV and V=80 mV. (d) An individual GluR2-lacking AMPA channel, permeable to calcium ions, exhibits inward rectification (red curve). The apparent conductance and reversal potential are not applicable in this case, but the latent conductance and reversal potential successfully reproduce the I-V curve, as illustrated by three cases (black dashed lines) at V=−80 mV, V=0 mV and V=80 mV. (TIF) [file pcbi.1012883.s004.tif]

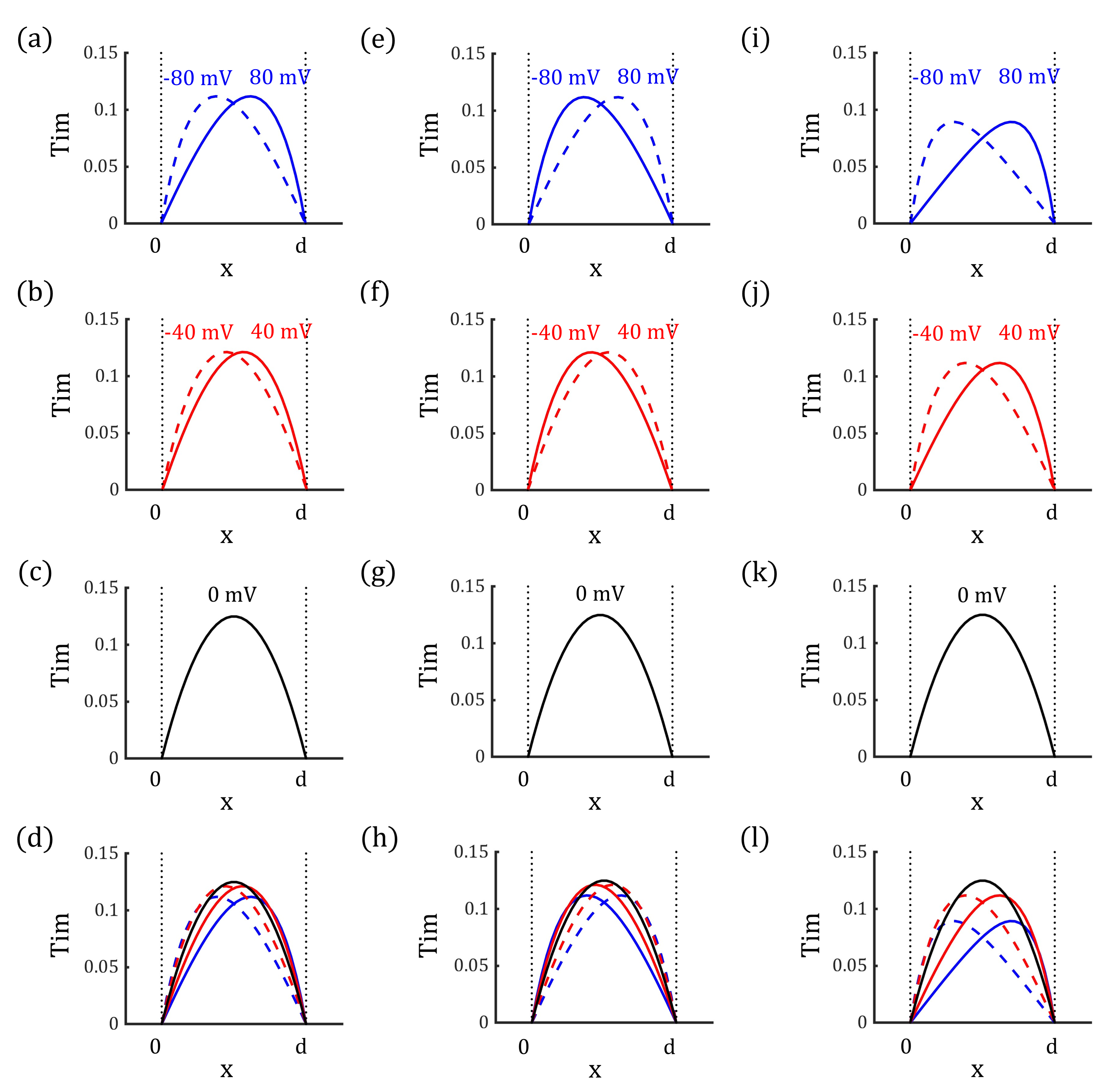

Supplement: S2 Fig — (a-d): For zq=+1. (e-h): For zq=−1. (i-l): For zq=+2. The function Tim reaches its maximum value of approximately 0.125 when V=0 mV and x=d/2. (TIF) [file pcbi.1012883.s005.tif]

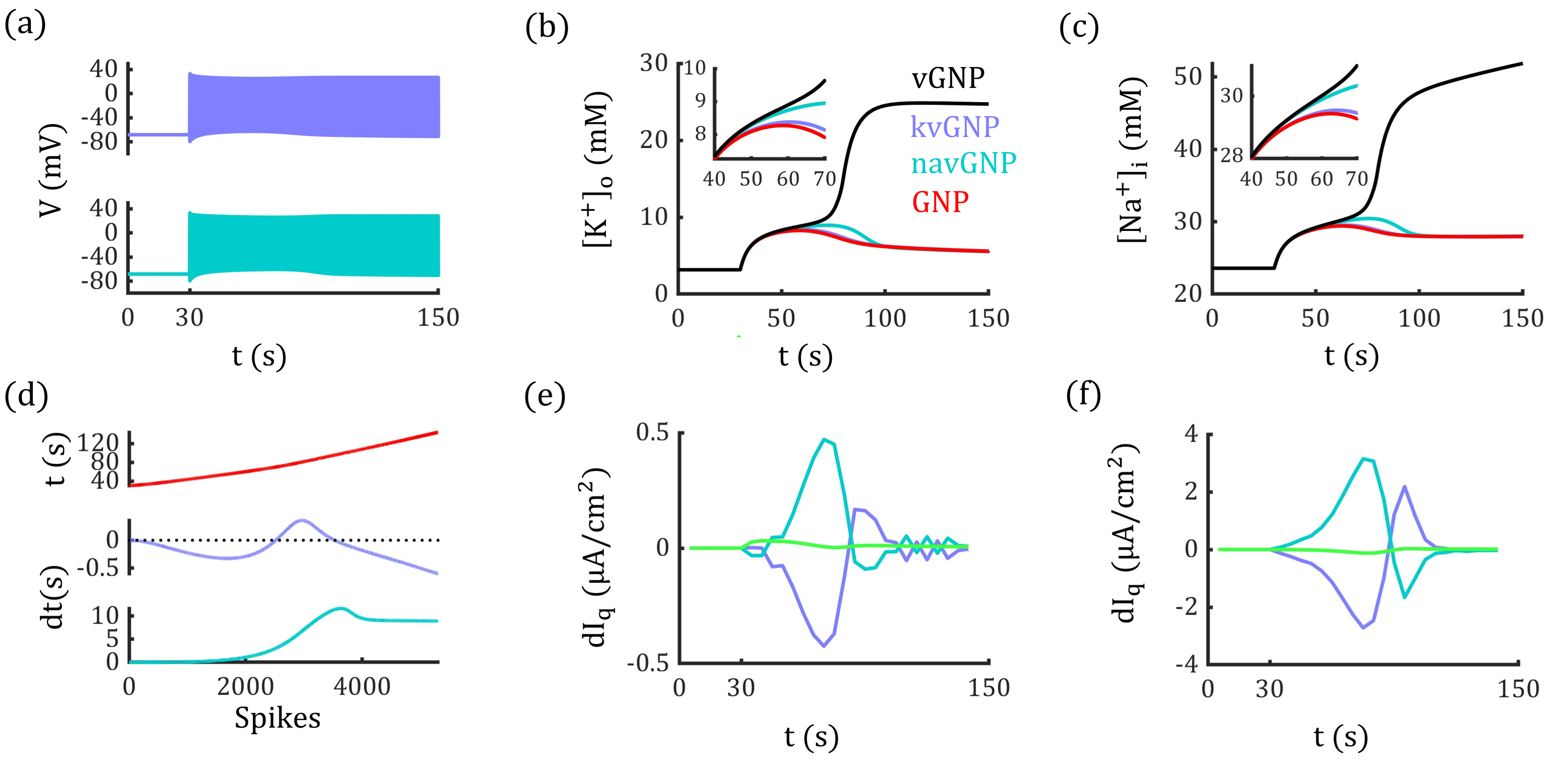

Supplement: S3 Fig — (a) Neural discharge events in kvGNP (upper panel) and navGNP (lower panel) models. The glutamate stimuli are identical to those used in Fig 8d (po=0.17). Both models exhibit tonic-spiking rather than entering a DB state. (b, c) Changes in extracellular potassium (b) and intracellular sodium (c) concentrations for four different models. Insets: zoomed in version of concentration changes between 40 s and 70 s. (d) Timing differences in neural discharge events in kvGNP (middle panel), navGNP (lower panel) models relative to the GNP model, illustrating the lag and lead in action potential timing. Upper panel: Timing of discharge events in the GNP model. (e, f) Differences in electrodiffusive potassium, sodium, and chloride currents in kvGNP (e) and navGNP (f) models, relative to the GNP model. Note that in the kvGNP model, the potassium current can be less inhibitory than in the GNP model, while in the navGNP model, the sodium current may be less excitatory during certain periods. (TIF) [file pcbi.1012883.s006.tif]
